# A randomized, controlled trial of a web-based tailored intervention to increase human papillomavirus vaccination among people living with HIV/AIDS

**DOI:** 10.1371/journal.pone.0319646

**Published:** 2025-03-31

**Authors:** Kaliane Caldas de Brito, Miralba Freire de Carvalho Ribeiro da Silva, Cristiane Wanderley Cardoso, Luciano Kalabric Silva, Ricardo Khouri, Antônio Eduardo de Albuquerque Junior, Nelzair Araújo Vianna, Maria da Conceição Chagas de Almeida, Edson Duarte Moreira Junior

**Affiliations:** 1 Gonçalo Moniz Institute, Oswaldo Cruz Foundation, Brazilian Ministry of Health, Salvador, Bahia, Brazil; 2 Assistência e Pesquisa, Centro Estadual Especializado em Diagnóstico, Salvador, Bahia, Brazil; 3 Secretaria Municipal da Saúde, Salvador, Bahia, Brazil; 4 Clinical Research Center, Charitable Works Foundation of Sister Dulce, Salvador, Bahia, Brazil; University of Ibadan, NIGERIA

## Abstract

**Background:**

Human papillomavirus (HPV) causes several cancers that disproportionally affect people living with HIV/AIDS (PLWH) yet there is a paucity of research on interventions to foster HPV vaccine use in this population. We sought to evaluate the efficacy of a web-based, tailored intervention (e-HPV) to promote HPV vaccination among PLWH.

**Methods:**

This is a randomized controlled trial with PLWH aged 18 to 45 years. Participants were recruited between January and June 2022 and randomized into two groups: experimental group (e-HPV), which received information about HPV and the HPV vaccine, based on the Protection Motivation Theory and control group, who received a clipping of information from the page maintained by the Ministry of Health dedicated to informing the population about HPV and the HPV vaccine. The primary and secondary outcomes were the percentage of PLWH willing to get HPV vaccine and HPV vaccine initiation (i.e., receipt of any doses by PLWH), respectively.

**Results:**

A total of 654 individuals were randomly allocated: 327 in the e-HPV and 327 in the control group. The average age was 29.7 years, the majority were men (71.4%), black or mixed race (63.2%). The intention to get vaccinated against HPV was approximately twice as high among participants in the e-HPV vs. control group (OR = 2.0, 95% CI: 1.3–3.4; p < 0.003), and HPV vaccine initiation was also significantly more common among participants in the e-HPV group (OR = 2.1, 95% CI: 1.1-4.0; p = 0.03). Belief in the effectiveness of the HPV vaccine, risk perception and the severity of an HPV infection were the reasons most reported by participants intending to get HPV vaccination.

**Conclusions:**

The intervention was acceptable and efficacious in increasing HPV vaccination among PLWH. Future studies are warranted to optimize and disseminate the e-HPV intervention to settings providing services to PLWH.

**Trial registration:**

Brazilian Clinical Trials Registry RBR-557mbvy

## Introduction

Human papillomavirus (HPV) is the most common sexually transmitted viral infection worldwide, and almost all sexually active people will be infected at some point in their lives [1,2]. The risk of acquiring HPV is approximately twice as high in the presence of human immunodeficiency virus (HIV) infection [3]. In addition, compared to the general population, people living with HIV/AIDS (PLWH) have a considerably increased risk for all types of HPV-associated anogenital cancers [3–5]. HIV-positive women have a 6-fold increased risk of developing cervical and vulva/vagina cancer compared to HIV-negative women [6,7]. HIV-positive men have a 38-fold increased risk of developing anal cancer compared to HIV-negative men and this risk is considerably higher among men who have sex with men (MSM) [7–9]. Coinfection with HIV and HPV appears to have multiple interactions related to altered immunity, increased susceptibility and possibly reactivation of latent HPV infection [10,11]. Genital warts are also more common and tend to recur and risk progression to malignant forms in HIV-positive people compared to HIV-negative people [12,13].

Currently, there are six HPV vaccines licensed and recommended by the World Health Organization (WHO) [14], they are considered effective and safe for the prevention of lesions caused by HPV among PLWH. Studies carried out among PLWH of different ages showed that, in general, these vaccines have seroconversion rates between 85% and 99% [15–20]. In Brazil, the Ministry of Health (MS) offers the quadrivalent vaccine (Gardasil©) free of charge for PLWH between 18 and 45 years. Despite this, coverage rates with the full scheme of this vaccine are below 50% [21,22].

Few studies address the issue of vaccination coverage among PLWH. A study carried out in the city of Varginha, Brazil showed HPV vaccine coverage of 28% among PLWH between 2007 and 2020 [23]. Other studies carried out in Brazil among PLWH indicate an unsatisfactory vaccination coverage for several immunizers [24,25]. Numerous barriers to HPV vaccination have been identified, such as lack of knowledge about the vaccine and absence/low perception of the risk of HPV infection [26–29]. However, it is not well established how relevant are these factors among PLWH. Healthcare providers often report inappropriate knowledge or experience to effectively manage complex medication regimens and the possibilities of multiple comorbidities in patients already living with HIV [30,31].

HPV vaccination social media-driven interventions have been efficacious in increasing HPV vaccination uptake and completion[32–34]. In addition to the low cost, electronic interventions have great dissemination capacity [35]. However, among PLWH, studies that involve social media-driven interventions are scarce. One such study found that text message reminder-recall improved HPV immunization uptake in young, HIV-1 infected patients [36]. More initiatives directed to improve vaccination among PLWH are needed. Thus, we developed a web intervention (e-HPV), targeted at mobile devices, containing information about HPV and the HPV vaccine to promote vaccination among PLWH. The current report present findings from a randomized controlled trial (RCT) on the efficacy of e-HPV on increasing HPV vaccine uptake and participants’ willingness to vaccinate.

## Materials and methods

This study was a randomized controlled single-blind trial (RCT) to evaluate an online tailored intervention to promote HPV vaccination among PLWH. Women and men between the ages of 18–45 years, living with HIV/AIDS, and who had not received any dose of HPV vaccine were eligible to participate. They were randomized via an automated process on the project website with a 1:1 allocation ratio into either the intervention or control group until the target sample size was reached.

A convenience sample of PLWH was recruited from January 6^th^ to June 30^th^ 2022. via three different approaches: i) printed invitations on leaflets available at a main public HIV outpatient health service (Centro Estadual Especializado em Diagnóstico, Assistência e Pesquisa – CEDAP, Salvador, Brazil); ii) invitations posted online in social media (Instagram) of influencers hosting content of interest for PLWH; and iii) invitations shared by support groups for PLWH or study participants. The invitations in all these three different approaches contained a specific link to direct the subject to the study website and to allow for identification of the participant recruitment source. The study website was hidden from web search engines to prevent access from other origins.

Eligible individuals’ participation was contingent upon the completion of informed consent online on the study website before enrollment. The study protocol and all the procedures were reviewed and approved by the Institutional Review Board (IRB) at the Gonçalo Moniz Institute (CAAE: 43282821.9.0000.0040).

The authors confirm that all ongoing and related trials for this intervention are registered at The Brazilian Clinical Trials Registry (No. RBR-557mbvy). This study was registered after patient recruitment began, as it is not a legal requirement for clinical trials conducted in Brazil to be registered. Nonetheless, the research protocol version registered was the same one the IRB had previously approved before study enrollment started. All data were collected electronically through a secure, encrypted connection, protected by a firewall and saved in a database with restricted access.

### Intervention and control materials

The intervention website (e-HPV) content about HPV and HPV vaccination was developed based on the protection-motivation theory (PMT) by epidemiologists (NVA, MCCA, and EDM) at the Gonçalo Moniz Institute, Oswaldo Cruz Foundation. The design of e-HPV, including the layout, appearance, and content (e.g., imagery, infographics, messages) was targeted for PLWH and built by health communication specialists and website designers (S1 Addendum). Then, the e-HPV material was revised and individually tailored by a focal group comprised of PLWH and specialists (n =  20), who assessed the website and intervention content for acceptability, clarity, and relevance, with changes made for its refinement.

The e-HPV was built with sequential sections, reached by “rolling” the screens as in many popular mobile apps (available at the following link: https://projetoihpv.bahia.fiocruz.br). There were four sections as follows:

1. *What is HPV?* this included epidemiological information about the prevalence and transmission of HPV, and HPV-related diseases among PLWH. We used two theoretical constructs perceived severity and perceived vulnerability.
2. *HPV vaccine*: provided information about HPV vaccine recommendations for PLWH and vaccine effectiveness, and individually tailored testimonials illustrating one’s reasons for getting vaccinated. The theoretical construct was the response efficacy, in this case vaccination.
3. *Questions & answers:* provided information to address potential barriers and concerns about HPV and HPV vaccination frequently reported in previous surveys [28,33,37,38], in our focal group, using a question-and-answer format.
4. *How to get vaccinated:* provided information on the logistics of getting the HPV vaccine (the response cost), including local and time for vaccination services, as well as telephone numbers to contact reference centers for special immunobiologicals (Centros de Referência para Imunobiológicos Especiais – CRIE), where HPV vaccination is offered at no cost for PLWH.

The control website content was adapted from the Brazilian Ministry of Health website on HPV prevention and vaccination [39]. The material was presented in the same format as the e-HPV and included information about HPV and HPV-related disease, health benefits of HPV vaccination, current HPV vaccine recommendations, and the safety of HPV vaccination (S1 Addendum). Materials for both groups (e-HPV and control) were delivered via a mobile-friendly website accessible by desktop/laptop, tablet computer, or smartphone.

### Data collection

We assessed willingness to get HPV vaccine asking each participant the following question in the pre- and post-website engagement timepoint: “Would you get the HPV vaccine?”, the answers were “Yes”, “No” or “Maybe”. All participants who did not complete this question had “no” assigned as their responses. In addition, participants were also asked at the post-website engagement timepoint “Are you definitely getting vaccinated in the next 2 months?”. After engaging with the web-based content (either intervention or control materials), participants were asked to indicate why they were willing (or not) to get vaccinated from a list of commonly reported reasons [28,33,37,40], a subject could choose more than one motive. Besides, participants who consented and provided their e-mail address had a follow-up survey at 2 months after website engagement, when we gathered self-reported HPV vaccination data to measure HPV vaccine initiation (receipt of at least one dose of the HPV vaccine series).

The acceptability of study materials in both groups was also assessed by five agree-disagree items on a post-website engagement survey. Questions asked about participants’ perceptions regarding the quality of the information, quality of the website, and usefulness. We coded the items one to five, so that higher values denote greater acceptability. In the last part, participants provided data on demographic, health-related characteristics, and how often they used the internet to seek health information.

Sample size calculations indicated that we would need at least 586 subjects participating in the study to provide 80% power to detect a 10% or greater difference in HPV vaccination willingness between the two arms. This number assumes a control group rate of 20%, and a significance level of P < .05.

### Data analysis

The descriptive statistics of the sociodemographic and health-related characteristics were examined, however, as suggested for randomized trials, we did not compare study groups on these baseline characteristics with statistical tests [41,42]. Logistic regression models were applied to produce unadjusted odds ratios (ORs) with 95% confidence intervals (CIs) and compare study groups on willingness to get HPV vaccine and HPV vaccine initiation. After this primary analysis, we also assessed effect modification, that is if differential intervention efficacy occurred across demographic and health characteristics, by including in the logistic regression models an interaction term between each potential moderator and study group. Moderation was considered to be present if an interaction term had p < 0.05. Lastly, we used chi-square tests to examine differences for categorical variables, and independent sample t-tests to compare study groups on participant acceptability of website materials. All analyses were intent-to-treat and used two-tailed statistical tests with a critical alpha of 0.05. We used Stata version 14.0 (StataCorp LP, College Station, TX).

We adopted the recommendations of the Consolidated Standards of Reporting Trials (CONSORT) [41,42] statement and the Checklist for Reporting Results of Internet E-Surveys (CHERRIES). The calculation of the completion rate was based on navigation, since CHERRIES was designed for online questionnaires, and this study is a web intervention focused on navigation through the produced content, the questionnaire being a complement of data collection. The participation rate (or recruitment rate) followed the CHERRIES concept and was calculated by the ratio between randomized participants and those eligible for randomization.

## Results

**Fig 1:**
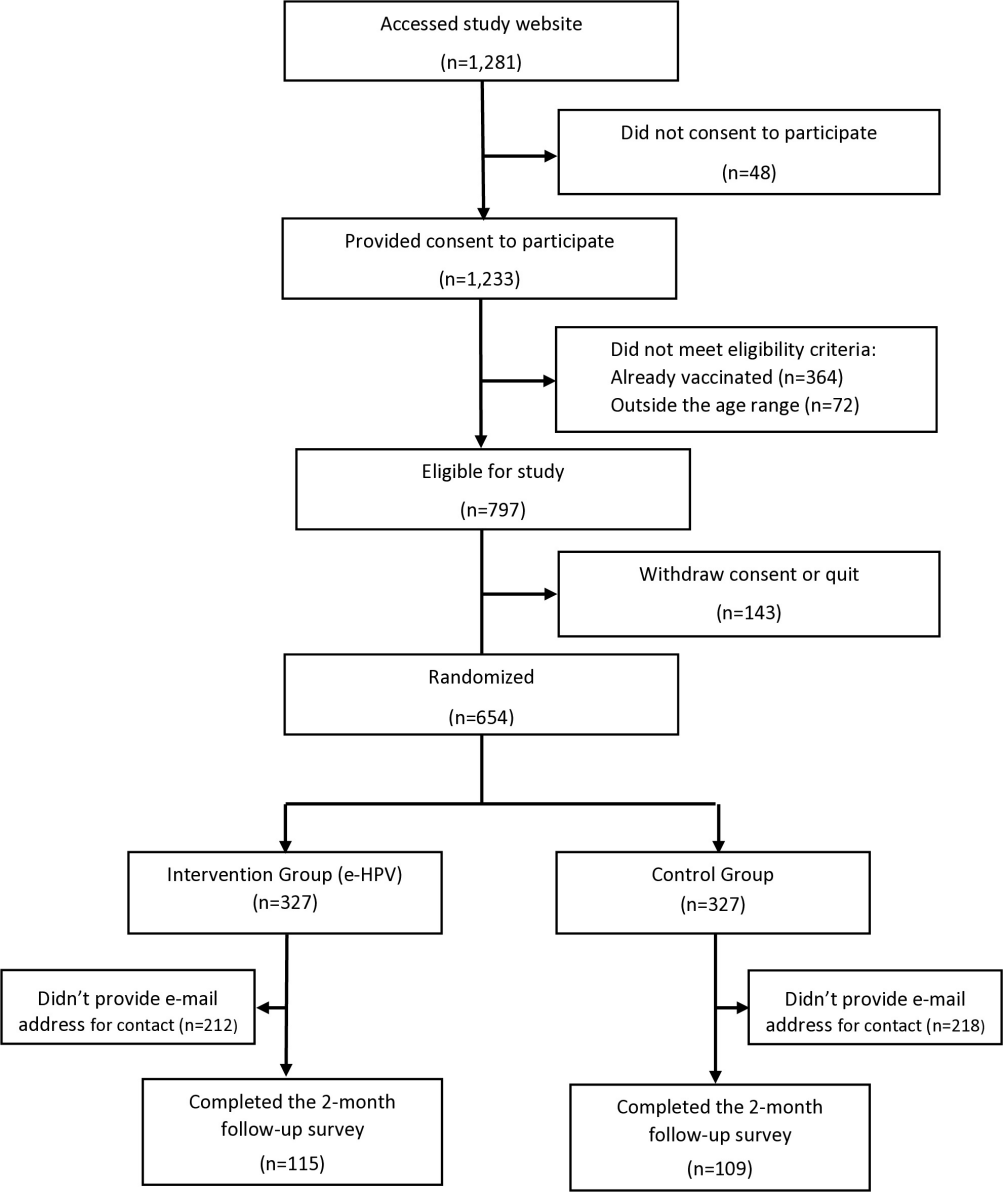
Flow diagram for the e-HPV intervention.

There were 1281 visitors to the study website, and 797 (62%) were eligible to participate. Of those, 654 were randomized, engaged in the website navigation, and completed the study survey for a response rate of 82% (Fig 1). A similar proportion of respondents provided an email address and consented to participate in the 2-month follow-up survey in the intervention group 35.2% (115/327) and in the control group 33.3% (109/327). Subjects who provided an email address and those refusing to do so were not significantly different in regard to the distribution of sociodemographic variables such as age, gender, and educational attainment.

Table 1 presents data on sociodemographic and selected health-related characteristics of study participants. Most were males (71%), aged 25 to 31, self-identified as white or mixed, single, and had some College education (or more). All three recruitment strategies were successful, and social media (Instagram) was the main source of recruitment (61%). Participants were from 22 out of 26 Brazilian States in all five regions. They often used the internet to seek health information, and one quarter had private insurance. Only 12% had ever received a doctor’s recommendation for HPV vaccination.

**Table 1:** Characteristics of participants by study group (n = 654), Brazil, 2022.

	Control group (n = 327)		Intervention group (n = 327)		Total (n = 654)	
	n	(%)	n	(%)	n	(%)
Age (years)						
18 - 24	100	(31)	74	(23)	174	(27)
25 - 31	111	(34)	115	(35)	226	(35)
32 - 38	75	(23)	92	(28)	167	(26)
39 - 45	41	(13)	46	(14)	87	(13)
Gender identity						
Male	237	(72)	230	(70)	467	(71)
Female	82	(25)	86	(26)	168	(26)
Other^a^	8	(2)	11	(3)	19	(3)
Race/color (n = 465)						
White	89	(35)	71	(33)	160	(34)
Mixed	84	(33)	76	(36)	160	(34)
Black	75	(30)	59	(28)	134	(29)
Other^b^	4	(2)	7	(3)	11	(2)
Relationship status (n = 471)						
Single, never married	177	(69)	148	(69)	325	(69)
Married or living with partner	58	(23)	57	(27)	115	(24)
Other^c^	21	(8)	10	(5)	31	(7)
Educational attainment (n = 471)						
Primary (some or complete)	33	(13)	9	(4)	20	(4)
High-school (some or complete)	91	(36)	86	(40)	177	(38)
Some College or more	131	(51)	121	(56)	252	(54)
Recruitment source						
Social media (Instagram)	215	(66)	187	(57)	402	(61)
CEDAP	60	(18)	88	(27)	148	(23)
Support groups and shared invitations	52	(16)	52	(16)	104	(16)
Use internet to seek health information (n = 473)						
Never or rarely	49	(19)	41	(19)	90	(19)
Always	208	(81)	175	(81)	383	(81)
Have private health insurance	82	(25)	78	(24)	160	(24)
Ever received a doctor’s recommendation for HPV vaccination	37	(11)	42	(13)	79	(12)

Note: percentages may not total 100% due to rounding. Intervention group = e-HPV.

CEDAP=State Center Specialized in Diagnosis, Assistance and Research.

^a^Other = Trans Women, Trans Man, Crossdresser, non-binary.

^b^Other = Asian, indigenous.

^c^Other = divorced, separated or widowed.

The willingness to get HPV vaccination and HPV vaccine initiation by study group is shown in Table 2. In general, intention to get HPV vaccinated was reported more often among participants in the e-HPV group compared to those in the control group (RR = 1.1, 95% CI: 1.0-1.2; p = 0.002). This difference between groups remained true regarding planning to get vaccinated soon (next two months), RR = 1.2, 95% CI: 1.1-1.3; p = 0.006). In the 2-month follow-up survey, 22% of participants (49/224) said they had initiated the HPV vaccine series. HPV vaccine initiation was more often reported by subjects in the e-HPV group compared to those in the control group (28% vs. 16%; RR = 1.8, 95% CI: 1.1-3.0; p = 0.027). Additionally, the risk difference showed that 12% (95% CI 1.6, 22.9) of subjects would begin HPV vaccination under e-HPV intervention that they would not have under control group. We have identified no effect modification of the demographic and health characteristics on the intervention efficacy.

**Table 2:** Willingness to get HPV vaccine and HPV vaccine uptake by study group.

	Control group		Intervention group (e-HPV)		RR	(95% CI)	p-value
	%	(n/Total)	%	(n/Total)			
Intention to get HPV vaccine:							
“I would get the HPV vaccine.”	83	(272/327)	91	(298/327)	1.1	(1.0, 1.2)	**0.002**
“I will definitely get vaccinated in the next 2 months.”	63	(207/327)	73	(240/327)	1.2	(1.1, 1.3)	**0.006**
HPV vaccine uptake at 2 month follow-up:							
Initiated HPV vaccination (at least 1 dose)	16	(17/109)	28	(32/115)	1.8	(1.1, 3.0)	**0.027**

RR= Relative risk, CI= confidence interval.

The motivations for willing to get HPV vaccine reported by participants after website engagement, regardless of study group, are shown in Table 3. The most common reasons included their perceptions about: HPV vaccine effectiveness, likelihood of contracting HPV infection, potential HPV vaccine protection against cancer and genital warts, and severity of HPV-related disease. Each of these constructs were reported by more than half of the study participants, whereas self-efficacy to get vaccinated was mentioned only by one fifth in both study groups. Participants who changed their mind about vaccination (i.e., those accepting HPV vaccination after the study intervention), compared to those already willing to vaccinate (i.e., study subjects accepting HPV vaccination before the intervention), reported reasons to get vaccinated based on similar constructs. Noteworthy, those changing their mindset about vaccination were more likely to cite perceived vulnerability to HPV infection as their main motivation to get vaccinated after the intervention irrespective of the study group they belonged to.

**Table 3:** Reasons for willing to get HPV vaccine reported by participants after the intervention in both study groups (n = 512).

	Already wanted to vaccinate (n = 453)^a^	Changed their mind about HPV vaccination (n = 59)^b^	*p-value* ^c^
“I believe in the effectiveness of the HPV vaccine.”	65^d^	59	0.4
“I feel at risk of contracting HPV infection.”	57	75	**0.01**
“The HPV vaccine protects against cancer and genital warts.”	56	51	0.5
“HPV infection can be serious.”	53	56	0.6
“I won’t have any difficulty getting vaccinated.”	22	19	0.6

Showing the 5 most common responses overall. Participants could select more than more than one reason from a predefined list. All other reasons were reported by fewer than 10% of participant.

^a^ Reported “Yes” about intention to get HPV vaccine before and after the intervention in both study groups.

^b^ Initially reported “No” or “Maybe” about intention to get HPV vaccine but changed to “Yes” after the intervention in both study groups.

^c^ Chi-square test.

^d^ Percentage.

Table 4 compares the acceptability of control and intervention website materials. Overall, participants in both groups considered their study materials highly acceptable, as they rated the quality measures with mean scores greater than 4.5 for all the assessments. Nonetheless, participants evaluating the e-HPV material (intervention group) were more likely to fully endorse their content was relevant, and easy to use (all p < 0.02) than participants assessing the control material. In addition, participants in the intervention (vs. control) group more strongly endorsed that they liked how their content was presented (p = 0.01). The comparisons between e-HPV and control website for other measures indicates that the intervention was equally or more acceptable than the control. Furthermore, materials in both study groups were rated as helpful to decide about HPV vaccination.

**Table 4:** Acceptability of online content by study group.

	Control group (n = 248)		Intervention group (n = 206)		*p-value*
	Mean	(SD)	Mean	(SD)	
The information was relevant	4.84	0.50	4.93	0.31	**0.02**
The information was easy to understand	4.78	0.56	4.83	0.48	0.26
I liked how the content was presented	4.75	0.62	4.88	0.39	**0.01**
The information helped me decide about HPV vaccination	4.54	0.79	4.62	0.82	0.34
The website was easy to use	4.77	0.61	4.88	0.39	**0.01**
The website loaded quickly	4.74	0.67	4.77	0.56	0.67

All items assessed by study participants on a 5-point agree-disagree scale (1 = strongly disagree to 5 = strongly agree). *P*-values represent results from independent t-tests. Statistical tests were two-tailed with a critical alpha of 0.05. SD = standard deviation.

## Discussion

Although HPV vaccine is currently recommended for PLWH, its coverage remains low [30,43] and efforts are needed to increase HPV vaccine use in this group. We developed and tested the e-HPV, an innovative web-based intervention targeted to PLWH. The results of this RCT indicate that e-HPV is an effective strategy to promote willingness to get HPV vaccination and to increase actual uptake of HPV vaccine as measured by vaccine initiation (i.e., getting one or more doses of the vaccine) among PLWH. Efficacy was similar across demographic and health characteristics, suggesting that the effect of e-HPV did not differ across various subgroups of participants. An important future step is to find out the impact of e-HPV on increasing actual uptake of HPV vaccine as measured by vaccine completion (i.e., getting two or three doses of the vaccine, according to the age of initiation).

We used a conservative study design, which compared two competing HPV vaccination strategies rather than comparing e-HPV to a no-intervention control group, therefore making it harder to detect a difference in the intervention impact. Even so, our intervention showed a statistically significant effect on HPV vaccination willingness and HPV vaccine initiation. This suggests that it is indeed advantageous to tailor interventions to improve HPV vaccination, providing more than basic education about HPV and HPV vaccine and adding other components. It’s been shown that multi-component interventions may be more effective than those using a single-pronged strategy[44]. The e-HPV is a bundled multiple components providing more than HPV education, as it also addresses targeted and tailored contents, self-efficacy strategies, and resources for getting HPV vaccination. All of these can influence key drivers of one’s decision on getting vaccinated.

Participants willing to get HPV vaccine in both study groups reported their most common motivations were based on similar constructs (i.e., perceptions of HPV vaccine effectiveness, perceived likelihood of contracting HPV infection, potential HPV vaccine protection against cancer and genital warts, and perceived severity of HPV-related disease). Of note, those changing their mindset about vaccination reported perceived vulnerability to HPV infection as their main motivation to get vaccinated after the intervention in both study groups. This provides further support to include tailored information about increased HPV-related diseases in PLWH. Our data showed that some theoretical constructs seem to impact the decision to get vaccinated and may act as mediators. Despite the intervention targeted HPV vaccination self-efficacy strategies, this construct was reported less often. Vaccination is not covered by private insurance in Brazil, and even though HPV vaccination is offered to PLWH free of charge in the National Immunization Program, it is only available at Special Immunobiological Reference Centers (CRIE) [45]. There are relatively few numbers of such centers in relation to the sizable population of PLWH they should serve, scheduling a visit and getting vaccinated may require a lot of effort and demand time [46]. Thus, barriers and lack of access to public services that offer HPV vaccination may be harder to overcome and require more than the skills-building strategies and resources for getting HPV vaccine provided in our intervention [22].

The items regarding the acceptability of the study materials were highly rated by participants in both study groups. They found that both interventions were easy to understand, and endorsed their materials were helpful to decide about HPV vaccination. These results may be due to the control intervention adaptation from the Ministry of Health website information about HPV vaccine, which was designed, similarly to the e-HPV, to be easy to understand (targeting a wide audience with low literacy) and to help decision about HPV vaccination. Nevertheless, participants in the e-HPV intervention more strongly supported that their web materials had relevant information, as well as they were easy to use, and that they liked how the intervention website looked. The control material content was directed broadly to HPV vaccination in the general population. In contrast, e-HPV contained information tailored to PLWH, including increased risk of HPV-related diseases in this group, which may explain the participants’ perceptions of more relevance for these materials. The control intervention presented information mostly with text, whereas e-HPV was planned to present information through a variety of visual formats, including infographics, imagery, data visualizations like pie charts and bar graphs, being concise with minimal text to give an easy-to-understand overview of the topics. These differences in format may have looked more appealing and led to the higher acceptability rates among the PLWH comprised mostly by young adults. It is also arguable that information with graphical presentation may have a better recall as compared to text only, since well-constructed visual aids have been shown to be highly effective, fast, memorable, and ethically desirable means of risk communication [47]. In addition, the infographics and other visual formats were developed through extensive and iterative formative research, including input from PLWH at different stages of the intervention’s development.

### Strengths and limitations

This study used a rigorous randomized controlled design. We adopted the recommendations of the CONSORT statement to enhance the transparency and accuracy of reporting our research. Besides, our study was reported according to the CHERRIES statement to give readers a better understanding of the sample selection and to increase the usefulness of this report. Study limitations include the assessment of HPV vaccination reliance only among participants providing an email address. Nevertheless, the consent for the follow-up contact was balanced between both study groups, since it was obtained before randomization. Another limitation is the assessment of HPV vaccination reliance on self-report data. There may be considerable variation between self-reported vaccination status compared to medical records data, but a recent study has shown more favorable agreement statistics for HPV vaccination data [48]. Although the current HPV vaccination web-based intervention has provided information on vaccine uptake (HPV vaccine initiation), there is no data on vaccine completion (i.e., receipt of all 2-3 doses recommended for this age range) to support external validity needed to translate the intervention into real world implementation. However, additional follow-up assessments are planned to ascertain actual vaccine completion rates. Most participants were recruited through Instagram, which could limit the generalizability of the findings, although the demographic characteristics of participants in our study resembled those of PLWH from other national studies [49]. Future research should consider recruiting PLWH through other social media sites that may have different audiences (e.g., Facebook, Twitter).

## Conclusions

HPV vaccination remains a public health priority. The current trial has shown the acceptability and efficacy of a web-based strategy for increasing HPV vaccination specifically for PLWH. Given these promising results, an important next step is to further establish the efficacy of e-HPV to improve vaccine completion, as well as determine the mechanism by which the intervention may affect vaccination behaviors. Our findings support continued efforts to pursue population-targeted, individually tailored and web-based strategies to address the low levels of HPV vaccine uptake among PLWH. More research is needed to establish how to disseminate this intervention to settings that provide services to PLWH and to determine how e-HPV can be further improved for impact and sustainability. Further research may also adapt this approach to other priority populations at increased risk of either HPV-related disease or undervaccination.

## Supporting information

S1 Checklist Paper_e-HPV_CONSORT-2010_Checklist. (DOCX)

S1 File Projeto-submetido_IRB_Clean. (PDF)

S2 File Protocol-submitted to IRB_English_Clean. (PDF)
