# Supplementary material for: A randomized, controlled trial of a web-based tailored intervention to increase human papillomavirus vaccination among people living with HIV/AIDS
Source: PLoS One. 2025 Mar 31;20(3):e0319646. doi: 10.1371/journal.pone.0319646 (PMC11957270; doi:10.1371/journal.pone.0319646)
Supplement: S1 File — (PDF) [file pone.0319646.s002.pdf]

PROJETO DE PESQUISA

INTERVENÇÃO EM DISPOSITIVO MÓVEL PARA AUMENTAR A COBERTURA DA VACINA HPV EM PESSOAS VIVENDO COM HIV/AIDS

Instituição proponente: Instituto Gonçalo Moniz – FIOCRUZ. Endereço: rua Waldemar Falcão, no. 121, Candeal, Salvador-BA. CEP: 40.296-710

Equipe:

<u>Instituto Gonçalo Moniz - FIOCRUZ</u>

Dr. Edson Duarte Moreira Junior
Pesquisador responsável.
Chefe do Laboratório de Epidemiologia Molecular e Bioestatística (LEMB),
Lattes: http://lattes.cnpq.br/9530545028476035

Me. Kaliane Caldas de Brito
Doutoranda do curso de pós-graduação em Biotecnologia em Saúde e Medicina Investigativa e Tecnologista em Saúde do Instituto Gonçalo Moniz Email:
Lattes: http://lattes.cnpq.br/6425155220370314

Salvador – Bahia
2020

RESUMO

INTRODUÇÃO: Em comparação com a população em geral, pessoas que vivem com HIV/Aids (PVHA) têm um risco consideravelmente aumentado para todos os tipos de cânceres anogenitais associados ao HPV. Esse vírus é responsável por praticamente todos os casos de câncer cervical, de ânus e verrugas genitais, e pela maioria dos casos de cânceres de vagina, vulva, pênis e orofaringe, representando um importante problema de saúde pública. A vacina contra o HPV foi introduzida pelo Programa Nacional de Imunizações (PNI) em 2014, porém as taxas de cobertura são baixas. O conhecimento em relação à vacina e ausência/baixa percepção do risco de infecção são apontados como fatores importantes entre aqueles que recusam a vacina. **OBJETIVO**: Avaliar a viabilidade, a aceitabilidade e a eficácia de uma intervenção baseada em dispositivos móveis contendo informações sobre o HPV e a vacina HPV para promover a vacinação em PVHA. O desfecho primário a ser avaliado é a intenção em se vacinar. **MATERIAIS E MÉTODOS**: Será realizado um estudo de intervenção, tipo ensaio randomizado controlado com PVHA, divididas em dois grupos. A um dos grupos serão apresentadas informações sobre o HPV e vacina HPV, elaboradas sob as bases da Teoria da Motivação para Proteção (TMP) numa intervenção *web* desenvolvida para dispositivos móveis. Outro grupo receberá um recorte de informações da página mantida pelo Ministério da Saúde dedicada a informar a população sobre o HPV e a vacina HPV. Os participantes elegíveis são pessoas entre 18 e 45 anos, que vivem com HIV, consentiram em participar do estudo e não tenham tomado qualquer dose da vacina contra o HPV. A intenção em se vacinar será obtida pelo percentual de participantes que declararem que irão ou pretendem se vacinar nos próximos três meses. A qualidade da intervenção também será avaliada pelos participantes da pesquisa.

Palavras-chave: Papilomavírus Humano; Vacina HPV; Vírus da Imunodeficiência Humana

SUMÁRIO

1. INTRODUÇÃO 5

2. HIPÓTESE....... 7

3. REVISÃO DA LITERATURA....... 8

3.1 A ASSOCIAÇÃO ENTRE O HPV E MALIGNIDADES ANOGENITAIS E DE CABEÇA E PESCOÇO 8

3.1.1 Câncer cervical....... 8

3.1.2 Outros cânceres anogenitais: ânus, vulva, vagina, pênis....... 9

3.1.3 Cânceres de cabeça e pescoço 9

3.2 DOENÇAS ASSOCIADAS AO HPV EM PESSOAS VIVENDO COM HIV/AIDS 11

3.2.1 Câncer cervical....... 11

3.2.2 Câncer anal....... 12

3.2.3 Câncer de vulva/vagina 13

3.2.4 Câncer peniano....... 13

3.2.5 Câncer de cabeça e pescoço 14

3.3 A VACINA CONTRA O HPV....... 15

3.4 A TEORIA DE MOTIVAÇÃO PARA A PROTEÇÃO....... 18

3.5 O USO DE DISPOSITIVOS MÓVEIS PARA INFORMAÇÃO EM SAÚDE 19

4. OBJETIVOS 22

4.1 OBJETIVO GERAL: 22

4.2 OBJETIVOS ESPECÍFICOS....... 22

5. MATERIAIS E MÉTODOS....... 23

5.1 SELEÇÃO DOS PARTICIPANTES 23

5.2 CRITÉRIOS DE INCLUSÃO 23

5.3 CRITÉRIOS DE EXLUSÃO 23

5.4 A INTERVENÇÃO....... 24

5.5 O GRUPO CONTROLE 25

5.6 COLETA DE DADOS 25

6. PROPOSTA DE ANÁLISE 26

6.1 VARIÁVEIS 26

6.1.1 Viabilidade dos convites 26

6.1.2 Aceitabilidade....... 26

6.1.3 Eficácia....... 27

6.2 METODOLOGIA DE ANÁLISE DOS DADOS 27

6.2.1 Desfecho primário 27
6.2.2 Desfecho secundário 27
6.2.3 Tamanho da amostra 28
7. CRONOGRAMA 29
8. ORÇAMENTO 30
9. CONSIDERAÇÕES SOBRE A ÉTICA 31
9.1 AVALIAÇÃO DOS RISCOS E BENEFÍCIOS: 31
9.1.1 Riscos 31
9.1.2 Benefícios 31
10. REFERÊNCIAS 33

1. INTRODUÇÃO

O papilomavírus humanos (HPV) é um vírus que infecta o epitélio anogenital masculino e feminino, sendo a infecção viral mais comum do trato reprodutivo. A maioria dos indivíduos sexualmente ativos adquirirá a infecção pelo HPV em algum momento de suas vidas (WHO, 2019). Está bem estabelecida a associação causal entre HPV e câncer cervical e há evidências crescentes de que o HPV é um fator relevante em outros cânceres anogenitais (ânus, vulva, vagina e pênis), bem como em cânceres de cabeça e pescoço (ARBYN *et al.*, 2012; BOSCH *et al.*, 2002; BRUNI *et al.*, 2019; SCHIM VAN DER LOEFF *et al.*, 2014).

Estudos observacionais sugerem que coinfecções pelo vírus da imunodeficiência humana (HIV) e pelo HPV podem ter múltiplas interações (LOOKER *et al.*, 2018). A interação entre as duas infecções sexualmente transmissíveis (IST) parece estar relacionada à alteração da imunidade, aumento da suscetibilidade e possivelmente reativação da infecção latente pelo HPV (DENNY *et al.*, 2012). Mulheres vivendo com HIV/Aids possuem um risco 5 vezes maior de desenvolver câncer cervical e quase 6 vezes mais de desenvolver câncer de vulva/vagina quando comparadas com mulheres não infectadas pelo HIV. Já entre os homens, a chance de desenvolver câncer anal aumenta em 38 vezes (FRISCH, 2000). Infecções anais por HPV são quase universais entre homens que fazem sexo com homens (HSH) infectados por HIV (SCHIM VAN DER LOEFF *et al.*, 2014).

Existem mais de 100 tipos de HPV, dos quais pelo menos 14 são causadores de câncer. Os tipos de HPV de alto risco oncogênico são detectados em cerca de 90% dos casos de câncer anal e praticamente 100% dos cânceres cervicais, sendo os mais comuns os tipos HPV-16 e HPV-18, que juntos, são responsáveis por cerca de 70% de todos os casos de câncer cervical em todo o mundo (ABREU *et al.*, 2018; ARBYN *et al.*, 2012; BRUNI *et al.*, 2019). Os tipos de HPV não cancerígenos (especialmente os tipos HPV-6 e HPV-11) estão associados a até 90% das verrugas anogenitais (BRASIL, 2018; CDC, 2019).

Atualmente, dispomos de três vacinas consideradas muito eficazes e seguras para a prevenção das lesões causadas pelo HPV: a vacina quadrivalente, a bivalente e a vacina nonavalente, que diferem basicamente quanto aos tipos de HPV contra os quais confere proteção e são recomendadas pela OMS (WHO, 2017a). No Brasil, o Ministério

da Saúde (MS) disponibiliza a vacina HPV quadrivalente, que oferece proteção contra os 4 tipos (6, 11, 16 e 18) de HPV desde 2014, quando foi incorporada ao Sistema Único de Saúde (SUS). Inicialmente restrita a meninas, passou, a partir de março de 2015, a contemplar também a população feminina de 9 a 26 anos de idade, vivendo com HIV/Aids. Dois anos depois, em 2017, a população alvo foi ampliada novamente e atualmente abrange meninas de 9 a 14 anos, meninos de 11 a 14 anos, mulheres e homens de 9 a 26 anos de idade vivendo com HIV/Aids e indivíduos submetidos a transplantes de órgãos sólidos, de medula óssea e pacientes oncológicos. Para ambos os sexos, a meta
é vacinar 80% da população alvo, sendo administrada em 2 doses com intervalo mínimo de 6 meses entre elas. Nos casos de PVHA, o esquema vacinal é diferente, com administração de 3 doses, sendo a segunda dose aplicada após 2 meses e a terceira após 6 meses da dose inicial (BRASIL, 2018).

Apesar de a imunização ser um importante componente na prevenção de infecções oportunistas entre PVHA, a cobertura vacinal, sobretudo entre os adultos, é baixa e sua queda tem sido observada em diversos países, inclusive no Brasil. Em 2018, a cobertura vacinal acumulada da vacina HPV entre meninas foi de 70,3% e 49,9% para a primeira e segunda dose, respectivamente. Entre os meninos, a cobertura vacinal da primeira dose foi de 42,3% e de 20,1% para a segunda dose (BRASIL, 2019a). Dúvidas em relação à segurança e eficácia das vacinas estão entre as principais causas para a não atualização do calendário vacinal entre esse grupo. Porém, de modo geral a vacinação contra o HPV apresenta eficácia acima de 95% na prevenção de anormalidades cervicais e verrugas genitais (ANDRADE *et al.*, 2016).

A introdução da vacina contra o HPV nos Programas Nacionais de Imunização é considera complicada por diversos fatores, dentre eles a necessidade de interlocutores para atingir uma população-alvo tão diversa, a sensibilidade em torno de uma vacina que protege contra uma IST, além da necessidade de uma comunicação e mobilização social robustas para ajudar na aceitação, garantir a conclusão do plano e diminuir os custos relacionados com o rastreio dos casos de abandono (WHO, 2016). A dificuldade na criação de mensagens eficazes é apontada como um importante desafio a ser superado para a promoção da vacina contra o HPV:

> As informações precisas, técnicas, culturalmente apropriadas, práticas e motivacionais devem ser veiculadas de forma a serem facilmente entendidas por públicos diferentes em momentos diferentes (WHO, 2017b, P.50)

2. HIPÓTESE

Com o propósito ajudar a criar estratégias de comunicação que lancem um apelo à ação, este trabalho tem a seguinte **questão norteadora: Em que medida uma intervenção, baseada em dispositivos móveis, para promover a vacinação contra o HPV entre adultos jovens vivendo com HIV/Aids é mais eficiente do que as intervenções tradicionais, baseadas na *Web*, atualmente utilizadas pelo Ministério da Saúde para informar sobre o HPV e vacina contra o HPV para PVHA?**

Parte-se da hipótese de que as informações sobre o HPV e a vacina contra o HPV, quando apresentadas de forma concisa, em linguagem direta, com mais recursos visuais, menos texto e com o conteúdo elaborado sob as bases teóricas da Teoria de Motivação para Proteção (TMP) são mais eficientes para despertar a intenção em se vacinar. Sendo assim, o presente trabalho se propõe a avaliar a viabilidade, a aceitabilidade e a eficácia de uma intervenção em *website* (voltado para dispositivos móveis) contendo informações sobre o HPV e a vacina HPV para promover a vacinação em pessoas vivendo com HIV/Aids**.** Dessa forma, espera-se contribuir para o aperfeiçoamento das ações de divulgação para a promoção da vacina contra o HPV entre essa população chave.

3. REVISÃO DA LITERATURA

3.1 A ASSOCIAÇÃO ENTRE O HPV E MALIGNIDADES ANOGENITAIS E DE CABEÇA E PESCOÇO

O HPV é um vírus que infecta o epitélio anogenital masculino e feminino, sendo a infecção viral mais comum do trato reprodutivo (WHO, 2019). Ele forma um grupo altamente diversificado com mais de 100 tipos, os quais normalmente são agrupados pelo seu potencial oncogênico. Quanto a esse potencial, podem ser classificados como de baixo risco (tipos 6, 11, 42, 43 e 44) ou de alto risco (tipos 16, 18, 31, 33, 35, 39, 45, 46, 51, 52, 56,58, 59 e 68) (ABREU *et al.*, 2018; ARBYN *et al.*, 2012). A infecção pelo HPV causa, globalmente, mais de 600 mil casos de câncer de colo do útero, vulva, vagina, pênis, ânus e orofaringe a cada ano, além de doenças benignas, como verrugas genitais (MARTEL, de *et al.*, 2017).

3.1.1 Câncer cervical

HPV é causa necessária para o câncer cervical (BOSCH *et al.*, 2002). Os tipos HPV-16 e HPV-18 são responsáveis por cerca de 70% de todos os casos de câncer cervical em todo o mundo, sendo o HPV-16 sozinho, a causa de aproximadamente 50% de todos os casos (ABREU et al., 2018). O câncer cervical é um importante problema de saúde pública devido à alta incidência e mortalidade. É o terceiro câncer mais comum entre as mulheres, o segundo entre mulheres de 15 a 44 anos. Globalmente, estima-se em 569.847 novos casos e 311.365 mortes decorrentes do câncer cervical em 2018 (BRUNI et al., 2019). Mais de 85% destas mortes ocorrem em países de baixa e média renda, sobretudo em países africanos subsaarianos, que possuem o maior índice de HIV (WHO, 2019). No Brasil, o câncer cervical é a terceira causa de morte por câncer entre mulheres e faz, por ano, 5 mil vítimas fatais (BRASIL, 2018). São estimados em mais de16 mil casos novos para cada ano do biênio 2018-2019, com um risco estimado de 15,43 casos a cada 100 mil mulheres (INCA, 2017). Projeções da Agência Internacional de Pesquisa em Câncer mostram que, caso medidas preventivas não sejam tomadas e rapidamente implementadas, em 2040, o número de mortes causadas pelo câncer cervical sofrerá um aumento de 50% em relação a 2018 (IARC, 2019).

3.1.2 Outros cânceres anogenitais: ânus, vulva, vagina, pênis

Globalmente, a cada ano, aproximadamente 8.500 casos de carcinoma vulvar, 12.000 de câncer vaginal, 35.000 de câncer anal e 13.000 de câncer peniano são atribuídos ao HPV (MARTEL, de *et al.*, 2017).

O câncer anal é semelhante ao câncer do cervical em relação à positividade geral do DNA do HPV (Bruni *et al.*, 2019). Os tipos de HPV-16 e HPV-18 são responsáveis por 87% de todos os casos (MARTEL, de *et al.*, 2017), sendo o HPV-16 o mais prevalente (75%) (ABRAMOWITZ *et al.*, 2011). Quase 90% dos cânceres anais são atribuíveis ao HPV e, globalmente, a malignidade é igualmente distribuída nos dois sexos (MARTEL, de *et al.*, 2017). Raro na população em geral, com uma incidência média mundial de 1 por 100.000 pessoas (Bruni *et al.*, 2019), o câncer anal ocorre um pouco mais frequentemente em homens em países menos desenvolvidos e em mulheres em países mais desenvolvidos (MARTEL, de *et al.*, 2017). Ele também vem aumentando entre as populações de homens que fazem HSH, mulheres com história de câncer cervical ou vulvar e populações imunocomprometidas, incluindo aquelas infectadas pelo HIV (BRUNI *et al.*, 2019; SCHIM VAN DER LOEFF *et al.*, 2014).

O câncer de vulva é relativamente raro entre as mulheres em todo o mundo, com estimativa de 27.000 novos casos, em 2008. Em todo o mundo, cerca de 60% de todos os casos de câncer vulvar ocorrem em países mais desenvolvidos (BRUNI *et al.*, 2019). Os HPV-16 e HPV-18 são responsáveis por cerca de 72% dos casos, sendo 85% destes, atribuídos apenas ao HPV-16 (MARTEL, de *et al.*, 2017).

A incidência do câncer de vagina é estimada em 13.000 novos casos em 2008 (BRUNI *et al.*, 2019), dos quais 78% dos casos são atribuíveis aos HPV-16 e HPV18 (MARTEL, de *et al.*, 2017).

Globalmente, o câncer de peniano foi estimada em 22.000, em 2008 (Bruni *et al.*, 2019). Aos HPV-16 e HPV-18 são atribuídos 70% dos casos (MARTEL, de *et al.*, 2017). Nos tumores invasivos do pênis, o HPV-16 foi o tipo mais comum detectado (40%) (RUBIN *et al.*, 2001).

3.1.3 Cânceres de cabeça e pescoço

Três locais de câncer na cabeça e pescoço foram associados com HPV: orofaringe e, em grau muito mais fraco, cavidade oral e laringe (MARTEL, de *et al.*, 2017). Uma

metanálise mostrou que a prevalência geral do HPV em tumores de cabeça e pescoço aumentou significativamente ao longo do tempo: passou de 40,5% antes de 2000, para 64,3% entre 2000 e 2004 e 72,2% entre 2005 e 2009 (LEONCINI *et al.*, 2014).

Globalmente, aproximadamente 38.000 casos de câncer de cabeça e pescoço são atribuíveis ao HPV. Destes, cerca de 29.000 são casos de câncer de orofaringe (que incluem principalmente as amígdalas e a base da língua), 4.400 cânceres da cavidade oral e 3.800 casos de laringe (MARTEL, de *et al.*, 2017). O HPV-16 é apontado como o responsável por 96% dos casos de câncer de orofaringe. As evidências atuais sugerem que os tipos de HPV-16 e HPV-18 também estão associados com 85% dos cânceres de cabeça e pescoço (MARTEL, de *et al.*, 2017; WHO, 2017a).

A tabela 1 apresenta os principais tipos de câncer relacionados ao HPV, bem como os percentuais atribuíveis aos HPV-6, HPV-11, HPV-16 e HPV-18, presentes na vacina quadrivalente.

Tabela 1 – Percentuais atribuíveis de cânceres relacionados aos HPV-16/18 e HPV-6/11/16/18/31/33/45/52/58. Fonte: Adaptada de MARTEL, de *et al.*, 2017

**Cânceres relacionados ao HPV**	**Número atribuível ao HPV**	**Contribuição relativa dos HPV-16/18**		**Contribuição relativa dos HPV-6/11/ 16/18/31/33/45/52/58**	
		**Percentual**	**Número**	**Percentual**	**Número**
Cervical	530.000	70	370.000	89	470.000
Ânus	35.000	86	30.000	94	33.000
Vulva	8.500	73	6.200	87	7.400
Vagina	12.000	62	7.400	83	9.900
Pênis	13.000	70	9.100	85	11.000
Cabeça e Pescoço	38.000	84	32.000	89	34.000

Além dos casos de câncer descritos acima, o HPV está associado a verrugas anogenitais, sendo que os HPV-6 e HPV-11 estão associados a até 90% dos casos (WHO, 2019). Além disso, indivíduos com verrugas genitais têm um risco aumentado a longo prazo de desenvolver cânceres anogenitais e de cabeça e pescoço e estes riscos permaneceram elevados por mais de 10 anos após o diagnóstico de verrugas genitais (BLOMBERG *et al.*, 2012).

3.2 DOENÇAS ASSOCIADAS AO HPV EM PESSOAS VIVENDO COM HIV/AIDS

Em comparação com a população em geral, os indivíduos infectados pelo HIV têm um risco consideravelmente aumentado para todos os tipos de cânceres anogenitais associados ao HPV (FRISCH, 2000). As evidências atuais sugerem que as infecções por HIV e HPV podem interagir de várias maneiras. Além de infectarem locais anogenitais, são influenciados por fatores de risco semelhantes, como o número de parceiros sexuais. O comprometimento imunológico mediado pelo HIV causa aumento na aquisição e persistência do HPV (DREYER, 2018).

Uma meta-análise indicou que o risco de aquisição do HPV é aproximadamente o dobro na presença de infecção pelo HIV. Indicou também uma quase duplicação da aquisição do HIV naqueles indivíduos com infecção pelo HPV (LOOKER *et al.*, 2018). Um estudo entre mulheres no Zimbábue constatou que, dentro de 6 meses após um incidente com infecção pelo HIV, a positividade anogenital do DNA do HPV aumentou acentuadamente - mesmo sem um aumento no comportamento sexual de risco necessário para a infecção (NOWAK *et al.*, 2011). Dessa, forma a aquisição de HIV e HPV é aumentada pela infecção existente com o outro vírus, e o curso clínico da doença relacionada ao HPV é negativamente influenciado pela baixa imunidade (DREYER, 2018).

Pessoas infectadas com HPV têm maior risco de adquirir o HIV do que pessoas que não estão infectadas com HPV, mesmo após o ajuste para fatores de risco (HOULIHAN *et al.*, 2013). Já em áreas de alta prevalência de HIV, mulheres HIV positivas podem causar aumentos na prevalência de infecção genital por HPV em homens HIV negativos e, assim, aumentar o HPV circulando na comunidade (WILLIAMSON, 2015). Por outro lado, vários estudos transversais e longitudinais demonstraram que a supressão viral do HIV e maiores contagens de células T CD4 ajudam a reduzir a replicação do HPV, persistência e infecções múltiplas (KELLER *et al.*, 2012).

3.2.1 Câncer cervical

As neoplasias relacionadas ao HPV, especialmente o câncer cervical, causam morbimortalidade significativa entre os grupos infectados pelo HIV (DREYER, 2018). Em um estudo realizado em mulheres do Zimbábue, a infecção pelo HIV foi associada à depuração do HPV, indicando que a resposta imune ao HPV pode resultar em um aumento

de células suscetíveis ao HIV no trato genital. Assim, mulheres HIV positivas têm maior incidência e progressão mais rápida de câncer cervical (WILLIAMSON, 2015). Por conta da imunosupressão, além de apresentarem taxas mais altas de HPV e câncer cervical, são também resistentes ao tratamento de doenças relacionadas ao HPV e propensas ao desenvolvimento acelerado de câncer associado ao HPV (CHATURVEDI et al., 2009). Acredita-se que o HIV exacerba a carga do câncer do colo do útero (LOOKER et al., 2018).

Em Ruanda, um estudo conduzido entre mulheres com alto risco de IST, mostrou que a prevalência de qualquer HPV foi de 47% entre aquelas HIV-negativas, e de 72% nas mulheres soropositivas (VELDHUIJZEN et al., 2011). O intervalo entre a aquisição da infecção pelo HPV e a progressão para carcinoma invasivo é geralmente 20 anos ou mais (DENNY et al., 2012). No entanto, em mulheres com infecção por HIV não tratada, esse tempo é reduzido para de 5 a 10 anos (WHO, 2019). Um estudo que acompanhou mais de 50 mil mulheres vivendo com HIV/Aids nos Estados Unidos durante 5 anos, demonstrou que o câncer cervical invasivo ocorreu em excesso estatisticamente significativo entre as mulheres vivendo com HIV/Aids (RR 5,4; IC95% 3,9-7,2) (FRISCH, 2000).

3.2.2 Câncer anal

Infecções anais com HPV de alto risco são muito comuns entre indivíduos HIV positivos (ABRAMOWITZ et al., 2011), sobretudo entre HSH (SCHIM VAN DER LOEFF et al., 2014). É o que aponta o estudo de Frisch (2000) que também incluiu mais 250 mil homens vivendo com HIV/Aids e demonstrou que os cânceres anais invasivos e in situ ocorreram em excesso, principalmente entre os pacientes com menos de 30 anos, entre os quais os RRs estavam acima de 100. Entre os homens mais velhos, o risco aumentou significativamente de 20 a 40 vezes. Entre as mulheres, os RRs para câncer anal invasivo (RR 6,8; IC95% 2,7-14,0) e câncer anal in situ (RR 7,8; IC95% 0,2-43,6) foram tão altos quanto os cervicais e vulvar/vaginal. Embora elevados em todos os grupos étnicos, os RRs para o câncer anal foram maiores em brancos do que em negros ou hispânicos.

Uma revisão sistemática resumiu dados sugerindo que também entre homens heterossexuais infectados pelo HIV, a prevalência de HPV anal era maior do que entre aqueles HIV negativo (NYITRAY, 2012). Outros estudos também mostraram que a

prevalência de HPV anal era mais alta entre mulheres infectadas pelo HIV do que entre mulheres negativas para o HIV (SCHIM VAN DER LOEFF et al., 2014).

Um estudo prospectivo de mulheres maiores de 18 anos, atendidas em três centros clínicos para HIV, identificou que a neoplasia intraepitelial anal de baixo grau estava presente em 12% dos pacientes infectados pelo HIV e em 5% das mulheres não infectadas pelo HIV. A neoplasia intraepitelial anal de alto grau estava presente em 9% das mulheres infectadas pelo HIV e em 1% das mulheres não infectadas pelo HIV (HESSOL et al., 2009). Entre adolescentes, de 13 a 18 anos, as meninas também apresentam prevalência mais alta. Enquanto a prevalência de infecção anal por HPV nos meninos infectados pelo HIV foi 48% contra 36% entre os não infectados, nas meninas o número foi 59% entre as soropositivas para HIV e 13% entre as não infectadas (MOSCICKI et al., 2003).

A prevalência de HPV anal raramente foi mensurada entre mulheres adultas saudáveis que são soronegativas (NYITRAY, 2012). Mas um estudo de coorte realizado no Havaí com 1378 mulheres mostrou que 50% das participantes tiveram infecções por HPV anal (SHVETSOV et al., 2009) Em uma meta-análise incluindo 8 estudos de HSH HIV negativos, a prevalência geral de HPV foi de 37,2% (MACHALEK et al., 2012).

3.2.3 Câncer de vulva/vagina

Mulheres vivendo com HIV/Aids possuem um risco de desenvolver câncer de vulva/vagina quase 6 vezes maior quando comparadas com mulheres não infectadas pelo HIV. Na faixa etária de 0 a 29 anos, os RR foram de 37,2 (IC 95% 7,7 a 108,8) para lesões invasivas e 14,5 (IC 95% 4,0 a 37,1) para lesões in situ. (FRISCH, 2000). Muito embora (CHATURVEDI et al., 2009) tenham identificado um risco menor para o desenvolvimento de câncer de vagina ou vulva in situ em PVHA (RR = 1,52, IC 95% = 0,99 a 2,35, P = 0,055)

3.2.4 Câncer peniano

O câncer peniano *in situ* ocorrem em excesso entre homens vivendo com HIV/Aids (RR 6,9; IC95% 4,2-10,6), em particular, entre aqueles com menos de 30 anos (RR 16,1; IC95% 4,4–41,2). Considerando todas as faixas etárias juntas, os riscos de câncer invasivo e *in situ* de pênis aumentaram cinco vezes ou mais entre negros e

hispânicos (FRISCH, 2000). A tabela 2, a seguir, traz os riscos relativos por idade dos cânceres anogenitais associados ao HPV entre os 309.336 pacientes com HIV/AIDS que participaram do estudo.

Tabela 2 – Riscos relativos por idade dos cânceres anogenitais associados ao HPV entre os 309.336 pacientes com HIV/AIDS. Fonte: Adaptado de FRISCH, 2000

	Risco Relativo (95% intervalo de confiança)				
Idade	Cervical	Vulva/Vagina	Ânus (Mulheres)	Ânus (Homens)	Pênis
			**Câncer Invasivo**		
<30	6.1 (2.6–12.0)	37.2 (7.7–108.8)	134.3 (16.3–484.8)	162.7 (103.1–244.0)	37.2 (7.7–108.6)
30-39	5.9 (3.8–8.7)	8.5 (3.1–18.4)	12.2 (2.5–35.7)	40.1 (31.2–50.8)	7.4 (2.4–17.3)
40-49	6.0 (3.1–10.4)	3.0 (0.4–10.8)	2.8 (0.1–15.6)	39.3 (31.3–48.7)	2.2 (0.4–6.3)
>50	—†	1.7 (0.0–9.3)	2.4 (0.1–13.5)	23.4 (16.6–32.0)	1.8 (0.4–5.3)
Todas	5.4 (3.9–7.2)	5.8 (3.0–10.2)	6.8 (2.7–14.0)	37.9 (33.0–43.4)	3.7 (2.0–6.2)
			**Câncer in *Situ***		
<30	5.3 (4.6–6.1)	14.5 (4.0–37.1)	—	130.4 (71.2–219.0)	16.1 (4.4–41.2)
30-39	4.3 (3.9–4.8)	3.4 (0.9–8.7)	21.0 (0.5–117.1)	72.7 (55.5–93.6)	8.4 (3.6–16.5)
40-49	4.7 (3.9–5.7)	1.0 (0.0–5.8)	—	38.0 (24.3–56.2)	4.8 (1.3–12.4)
>50	4.1 (2.4–6.6)	5.1 (0.6–18.5)	—	40.2 (17.3–79.3)	4.6 (1.3–11.8)
Todas	4.6 (4.3–5.0)	3.9 (2.0–7.0)	7.8 (0.2–43.6)	60.1 (49.2–72.7)	6.9 (4.2–10.6)

v

3.2.5 Câncer de cabeça e pescoço

Nos Estados Unidos, há um excesso de aproximadamente 50% nos casos de câncer na cavidade oral entre PVHA em comparação com a população. Esse excesso aumenta para quase 70% entre aqueles que vivem há cinco ou mais anos com AIDS (BEACHLER *et al.*, 2014).

As verrugas são causadas pelo HPV de baixo risco e são mais prevalentes entre pessoas infectadas pelo HIV, mas seu tamanho pode não se correlacionar bem com a imunidade (PERNOT *et al.*, 2014).

Na população em geral, a infecção por HPV é consideravelmente mais alta em jovens e diminui com a idade (LEWIS *et al.*, 2018). Dados preliminares do projeto POP-Brasil – Estudo Epidemiológico sobre a Prevalência Nacional de Infecção pelo HPV mostraram que a prevalência estimada do HPV entre os jovens de 16 a 25 anos é de 54,6% (POP-BRASIL, 2017). O perfil de prevalência para HPV-16 é 53,2% e 15,8% para HPV-18 (BRASIL, 2018).

De acordo com dados obtidos da Secretaria de Vigilância em Saúde, em 2018, foram diagnosticados 43.941 novos casos de HIV e 37.161 casos de aids no Brasil. Entre os anos de 2008 e 2018, observou-se um incremento na taxa de detecção entre jovens com idades de 15 a 19 anos e 20 a 24 anos na ordem de 62,2% e 94,6%, respectivamente. Apenas esse grupo é estimado em mais de 115 mil pessoas, considerando o ano de diagnóstico entre 2008 e 2018. Já entre as mulheres houve queda na taxa de detecção em todas as faixas etárias nesse mesmo período (BRASIL, 2019b). A notificação compulsória da infecção pelo HIV se iniciou em 2014, o que, adicionada ao fator de subnotificações, dificulta uma análise epidemiológica mais rigorosa com relação às tendências da infecção no Brasil.

3.3 A VACINA CONTRA O HPV

A primeira vacina contra o HPV (vcHPV) foi licenciada em 2006 e, desde então, muitos países a incluíram em seus programas nacionais de imunização (WHO, 2016). Atualmente, existem no mercado três vacinas profiláticas contra o HPV:

i. A vacina quadrivalente Gardasil (Merck Inc.): licenciada pela primeira vez em 2006 e direcionada aos HPV-16/18 oncogênicos e também aos tipos de baixo risco HPV-6/11 que causam verrugas genitais.
ii. A vacina bivalente Cervarix (GlaxoSmithKline): licenciada em 2007, oferece proteção para os HPV-16/18.
iii. A vacina nonavalente Gardasil 9 (Merck Inc.): licenciada em 2014, confere proteção contra os HPV-6/11/16/18 e outros cinco tipos mais carcinogênicos (HPV-31/33/45/52/58)(MARTEL, de *et al.*, 2017)

Nenhuma das vacinas contém produtos biológicos vivos ou DNA viral e, portanto, não são infecciosas; elas não contêm antibióticos ou agentes conservantes. Além dos além dos tipos virais para aos quais se destinam, elas também diferem no que diz respeito à indicação e a posologia. A vacina não é recomendada durante a gestação (WHO, 2017).

Após um esquema de 3 doses, as vacinas quadrivalente e bivalente foram avaliadas e mostram-se altamente imunogênicas com as respostas imunes mais altas observadas em meninas de 9 a 15 anos (SCHILLER, CASTELLSAGUÉ e GARLAND, 2012). Ambas conferem proteção por cerca de 10 anos (SCHWARZ *et al.*, 2019). Avaliada em três estudos de Fases II/III, a vacina quadrivalente apresentou taxas de soropositividade de 97,6%, 96,3%, 100% e 91,4% para os tipos HPV-6/11/16/18,

respectivamente (NYGÅRD *et al.*, 2015). Quanto aos efeitos adversos, uma meta-análise que incluiu 14 estudos mostrou que os principais efeitos adversos relacionados à vacinação foram dor, eritema, edema e febre (COELHO *et al.*, 2015).

1.1.1. Eficácia, segurança e imunogenicidade da vacina contra o HPV em PVHA

Um estudo da vcHPV quadrivalente em adolescentes e adultos jovens com idade entre 13 e 27 anos infectados pelo HIV mostrou que a vacina é segura e bem tolerada, com uma taxa de soroconversão de 85% nos infectados pelo HIV e 91 % em indivíduos HIV negativos. Não foram relatados eventos adversos graves ou com risco de vida. O efeito colateral local mais comum foi a dor que ocorreu em 18,8% dos pacientes HIV negativos e em 32,6% dos pacientes infectados pelo HIV. Já o efeito colateral sistêmico mais comum foi dor de cabeça, relatada em 13,5% dos infectados pelo HIV e em 2,2% dos indivíduos HIV negativos (GIACOMET et al., 2014).

Também foram avaliadas a segurança e imunogenicidade da VcHPV bivalente em mulheres infectadas pelo HIV na África do Sul. A vcHPV foi considerada segura em mulheres HIV positivas assintomáticas com idades entre 18 e 25 anos e não foram identificados impactos na contagem de células T CD4, na carga viral do HIV ou no estágio clínico do HIV (DENNY et al., 2013)

Um estudo com 319 mulheres infectadas pelo HIV nos Estados Unidos, Brasil e África do Sul mostrou que a vcHPV quadrivalente é segura e imunogênica em mulheres infectadas pelo HIV com idades entre 13 e 45 anos. As proporções de soroconversão para os quatro tipos de HPV (6, 11, 16 e 18) em mulheres com contagem de células T CD4 acima de 350 células/μL foram de 96%, 98%, 99% e 91%, respectivamente, enquanto as mulheres com CD4 contagem de menor de <200 células/μL tiveram proporções de conversão de 84%, 92%, 93% e 75%, respectivamente (KOJIC et al., 2014).

Em um ensaio clínico com HSH HIV positivos, vacinados com três doses da vcHPV quadrivalente, a soroconversão foi observada para todos os tipos de HPV (6, 11, 16 e 18) foram 98%, 99%, 100% e 95%, respectivamente. Não foram observados efeitos adversos nas contagens de CD4 + e no RNA do HIV1 no plasma. Também não houveram eventos adversos de grau 3 ou superior atribuíveis à vacinação entre os 109 homens que receberam pelo menos 1 dose da vacina. (Wilkin et al., 2010).

Já em crianças e adolescentes infectados pelo HIV, os dados sobre imunogenicidade e segurança da vcHPV quadrivalente são escassos. Até o momento, poucos estudos foram realizados. No entanto, num estudo de uma coorte de crianças e

adolescentes infectados pelo HIV com idades entre 8 e 11 anos a soroconversão para todos os 4 antígenos foi superior 96% entre aquele que receberam a vcHPV quadrivalente. Além de imunogênica, a vacina também mostrou-se segura (LEVIN et al., 2010).

Os resultados de um estudo multicêntrico realizado com 99 mulheres com idades entre 16 e 23 anos, HIV positivas, indicaram que as respostas imunes à vcHPV quadrivalente são robustas e a vacina bem toleradas. As taxas de soroconversão foram de 100% para os HPV-6/11/16/18 entre os participantes em terapia anti-retroviral. As taxas variaram de 92,3% (para o HPV-18) a 100,0% (para o HPV-6) entre os participantes que não estavam em terapia anti-retroviral. Um evento adverso grave (fadiga) foi observado (KAHN et al., 2013).

A Austrália, onde a vacina quadrivalente foi implementada no PNI deste 2007 e cuja taxa de cobertura foi superior a 70% para as três doses na população alvo, hoje colhe os frutos da sua iniciativa: diminuição em 92% da prevalência dos tipos de HPV 16 e 18 entre as mulheres com idade entre 18 e 35 anos (MACHALEK et al., 2018); 90% de redução de verrugas genitais em mulheres de 12 a 17 anos e 73% em mulheres com idade 18 a 26 anos, além da redução de 38% em homens com idade entre 18 e 26 anos, proteção indireta da vacinação exclusivamente feminina (SMITH et al., 2015). Na Escócia também observou-se uma redução significativa nos diagnósticos de neoplasia intraepitelial cervical nas mulheres que receberam três doses da vacina em comparação com mulheres não vacinadas (POLLOCK et al., 2014).

Apesar de a literatura indicar o alto potencial da vacina na prevenção de neoplasia cervical e lesões genitais, a vcHPV tem gerado controvérsias que repercutem na aceitação e na adesão por parte dos usuários (SILVA, OLIVEIRA e GALATO, 2019).

No Brasil, um estudo realizado em 2002, avaliou os conhecimentos e atitudes sobre o HPV, o câncer do cervical e o exame de Papanicolau em 204 mulheres com idade entre 16-23 anos, atendidas em uma um hospital público. Os dados revelaram que 67% das participantes não sabiam que o HPV pode causar câncer cervical/verrugas e apenas 10% reconheciam que o HPV poderia levar ao câncer cervical (MOREIRA et al., 2006).

Mendes Lobão e colaboradores (2018) conduziram um estudo para avaliar a aceitação da vacina HPV em centros urbanos brasileiros após sua inclusão no PNI. Para filhas e filhos de até 18 anos de idade, a aceitação parental da vacina HPV foi de 92% e 86%, respectivamente. O trabalho também revelou que aqueles que recusaram a vacinação tinham menor probabilidade de saber que o HPV é sexualmente transmissível e que causa verrugas genitais. Além disso, prevenção do câncer cervical e verrugas

genitais foi citada com menos frequência pelos pais pesquisados como uma das razões para aceitar a vacina conta o HPV. Embora a aceitação se mostre alta, não há percepção da gravidade dos agravos para os quais a vacina oferece proteção.

Numa revisão sistemática realizada com o objetivo de caracterizar a receptividade à vacina contra o HPV e descrever as barreiras e os facilitadores dessa receptividade, foram identificados 11 facilitadores e nove barreiras à receptividade. O conhecimento em relação à vacina foi o destaque entre os facilitadores e a falta/inadequada informação relativa à vacina e a ausência/baixa percepção do risco de infecção foram as barreiras mais citadas (SILVA, OLIVEIRA e GALATO, 2019).

3.4 A TEORIA DE MOTIVAÇÃO PARA A PROTEÇÃO

A Teoria de Motivação para Proteção (TMP) formou a base teórica da intervenção. Segundo ela, diante de informações sobre uma ameaça à saúde, a motivação para se proteger surge da avaliação cognitiva da gravidade dessa ameaça (gravidade percebida), da percepção de probabilidade de ocorrência (vulnerabilidade percebida), junto com a crença de que uma resposta de enfrentamento recomendada pode prevenir efetivamente sua ocorrência (eficácia de resposta) (Rogers, 1975). Segundo Maddux; Rogers (1983), uma variável importante que pode afetar a aceitação de uma resposta de enfrentamento recomendada é a quantidade de trabalho envolvida na sua implementação (custo de resposta). Se um evento não for avaliado como grave, como provável, ou como se nada puder ser feito a seu respeito, então nenhuma motivação de proteção seria despertada e, portanto, não haveria nenhuma mudança nas intenções comportamentais (Rogers, 1975).

Inicialmente, a TMP postulava que a motivação para proteção – e, portanto, a mudança de atitude – era uma função multiplicativa desses três processos de mediação. Mais tarde, após sua revisão, a noção multiplicativa foi abandonada e incluído o processo cognitivo da autoeficácia, ou seja, a crença de que a pessoa tem capacidade de executar com sucesso a resposta de enfrentamento à ameaça. Assim, a gravidade percebida, a vulnerabilidade percebida e a eficácia da resposta, em interação com a autoeficácia, determinam as intenções (motivação de proteção) de se engajar em determinado comportamento (MADDUX; ROGERS, 1983). A TMP tem sido utilizada em diversos trabalhos relacionados à motivação para proteção em saúde (CAMERINI *et al.*, 2019;

GAINFORTH, CAO e LATIMER-CHEUNG, 2012; GAINFORTH e LATIMER, 2012; MCREE *et al.*, 2018)

Um estudo conduzido na Suíça com 554 pais de alunos do ensino médio de 13 a 15 anos, utilizou os princípios da TMP para identificar preditores da intenção dos pais de seguir as recomendações oficiais de vacinação contra a vacina tríplice viral. A eficácia da resposta (vacinação) mostrou estar diretamente relacionada à intenção dos pais de aderir às recomendações de vacinação tríplice viral (CAMERINI *et al.*, 2019). A TMP também foi utilizada para prever a intenção e o comportamento da vacinação contra hepatite B em uma população de imigrantes adultos na China. A vulnerabilidade percebida e eficácia da resposta foram fatores significativos que determinaram a aceitação da vacina contra a hepatite B (LIU *et al.*, 2016)

Um estudo examinou os determinantes teóricos das intenções de vacinação contra o HPV avaliando as construções da PMT e da teoria do comportamento planejado (TCP) em três grupos: mulheres universitárias, pais de filhas e pais de filhos. Os resultados indicam que as construções PMT e TCP preveem intenções para os diferentes grupos. O foco na eficácia da resposta da vacina, e não na gravidade percebida do HPV, foi mais eficaz em aumentar as intenções de vacinação entre todos os grupos. O foco na vulnerabilidade ao HPV foi eficaz apenas entre mulheres universitária e pais de filhos, e auto-eficácia foi preditora de intenções entre mulheres universitárias e pais de filhas (GAINFORTH, CAO e LATIMER-CHEUNG, 2012).

Um estudo piloto para testar uma intervenção de vacinação contra o HPV entre 150 jovens gays e bissexuais com idades entre 18 e 25, mostrou que o início da vacinação contra o HPV foi maior entre aqueles que receberam informações sobre o HPV produzidas com base nos construtos da TMP (45% vs. 26%). Esse mesmo grupo também registrou maior tendência na conclusão do esquema completo de vacinação (11% vs. 3%) (REITER *et al.*, 2018).

3.5 O USO DE DISPOSITIVOS MÓVEIS PARA INFORMAÇÃO EM SAÚDE

De acordo com a pesquisa TIC Domicílios de 2017, que mede a disponibilidade e o uso das Tecnologias de Informação e Comunicações (TIC) no Brasil e conduzida pelo Centro Regional de Estudos para o Desenvolvimento da Sociedade da Informação (Cetic.br), o número de usuários de Internet no Brasil chegou a 120,7 milhões, o que representa 67% da população com dez anos ou mais. Desses, quase a totalidade (96%)

usou a Internet pelo telefone celular, sendo que 49% deles utilizaram a rede apenas por meio desse dispositivo (CETIC.BR, 2018). O perfil de uso exclusivo pelo celular foi mais comum entre os usuários de classes D e E e de áreas rurais, refletindo uma realidade em que os cidadãos de baixa renda não possuem múltiplos dispositivos de acesso à Internet, como acontece no caso das classes A e B. A pesquisa também apontou que 77% dos usuários de Internet acessam redes sociais e 44% procurou informações relacionadas à saúde ou a serviços de saúde na rede em 2017.

Tozzi e colaboradores (2010) utilizaram motores de busca para comparar a qualidade das informações em sobre o HPV e da vacina HPV em páginas da *web*, tanto em inglês quanto em italiano. Pontuações nos domínios de credibilidade, conteúdo e design foram maiores em páginas de agências de saúde pública ou universidades. Alguns dos sites credenciados pela OMS tanto na Itália quanto nos Estados Unidos, que incluem informações sobre a imunização contra o HPV, não foram exibidos pelos motores de busca utilizados no estudo, o que sugere que páginas da *web* com informações de alta qualidade sobre a vacina contra o HPV podem não ser facilmente recuperadas por usuários comuns. A maioria das páginas da *web* exibidas nessa pesquisa eram sites particulares ou de provedores de notícias, os quais normalmente hospedam anúncios pagos ou objetivam lucro.

Um estudo realizado com HSH identificou, por meio de um grupo focal, que o uso crescente de estratégias móveis foi apontado como um facilitador para o acesso às informações de saúde disponibilizada *online* (FONTENOT *et al.*, 2016). Seguindo essa tendência, pesquisadores americanos realizaram uma intervenção com HSH com idade entre 18 e 26 anos com foco no uso de dispositivos móveis e identificaram que, em comparação com os participantes do grupo controle, os participantes da intervenção relataram maior percepção de que HSH possuem maior risco de câncer anal em relação a outros homens, maior autoeficácia de vacinação contra o HPV e menos danos percebidos pelo uso da vacina (MCREE *et al.*, 2018). Numa época em que a desinformação circula em grandes volumes em meios digitais, é de suma importância dispor de todos os meios para munir a população de informação útil e de qualidade a fim de melhor balizar as decisões quanto a aceitação das vacinas.

Um estudo de intervenção realizado para avaliar o conhecimento prévio e o papel da ação educativa sobre a atitude em relação à vacinação contra o HPV em 200 mulheres de 18 a 30 anos revelou que a atitude frente à vacina contra o HPV foi diferente no grupo que recebeu a ação educativa. Este grupo manifestou maior adesão incondicional à

vacina, enquanto aquele sem intervenção condicionou a aceitação da vacina à obtenção de mais informações ou à recomendação do médico que lhe assiste. Os pesquisadores identificaram que apenas 28,6% das participantes tinham a informação correta sobre a ação preventiva da vacina contra o HPV, sendo que a maioria (71,4%) acreditava que a vacina apresentava fins terapêuticos (PEREIRA *et al.*, 2016).

Ações educativas aliadas às tecnologias móveis e aos construtos que motivem adequadamente o indivíduo a buscar proteção para os agravos relacionados à saúde podem aumentar as chances de sucesso das intervenções de promoção de vacinação.

4. OBJETIVOS

4.1 OBJETIVO GERAL:

Avaliar a viabilidade, a aceitabilidade e a eficácia de uma intervenção baseada em dispositivos móveis contendo informações sobre o HPV e a vacina HPV para promover a vacinação em pessoas vivendo com HIV/Aids.

4.2 OBJETIVOS ESPECÍFICOS

4.2.1 Avaliar a eficácia da intervenção, estimando:

4.2.1.1 Percentual de indivíduos com intenção de se vacinar

4.2.1.2 Percentual de indivíduos que iniciaram a vacinação

4.2.2 Determinar a aceitabilidade da intervenção quanto a:

4.2.2.1 Qualidade da informação

4.2.2.2 Qualidade do *website*

4.2.2.3 Utilidade da intervenção

4.2.3 Analisar a viabilidade da divulgação da intervenção nas seguintes estratégias:

4.2.3.1 Distribuição de pôsteres/panfletos

4.2.3.2 *Posts* em mídia social

5. MATERIAIS E MÉTODOS

Trata-se de um estudo de intervenção, tipo ensaio randomizado controlado com PVHA, divididas em dois grupos. A um dos grupos serão apresentadas informações sobre o HPV e vacina HPV, elaboradas sob as bases da Teoria da Motivação para Proteção (TMP). Outro grupo receberá um recorte de informações da página mantida pelo Ministério da Saúde dedicada a informar a população sobre o HPV e a vacina HPV. Os materiais para ambos os grupos serão entregues por meio de dois *websites* dirigido para dispositivos móveis (*mobile friendly*) com identidade visual comum ao projeto. O *website* "i-HPV", que será exibido para o grupo intervenção e o *website* "MS-HPV", que será exibido para o grupo controle.

5.1 SELEÇÃO DOS PARTICIPANTES

O recrutamento será realizado por meio de duas estratégias: divulgação de convites impressos (pôsteres e panfletos) e convites *online* (*posts* em mídias sociais).

5.1.1 Convites impressos: Realizado pela disponibilização de panfletos e pôsteres em locais de assistência à população chave do estudo na cidade de Salvador, Bahia.

5.1.2 Convites online: Pela publicação gratuita de posts em mídias sociais de instituições e organizações de boa visibilidade e reputação voltadas para divulgação de pautas de interesse para PVHA.

5.2 CRITÉRIOS DE INCLUSÃO

i. Viver com HIV/Aids.
ii. Idade entre 18 e 45 anos.
iii. Consentirem em participar do estudo.

5.3 CRITÉRIOS DE EXLUSÃO

i. Ter tomado alguma dose da vacina HPV.

A alocação dos participantes será feita na proporção de 1:1, por meio de algoritmo computacional. Para que a ordem de alocação não seja descoberta, o algoritmo aloca, de forma aleatória os participantes em um dos grupos, porém, garantindo que a diferença do número de participantes em cada grupo não seja maior que cinco. Além disso, tendo em vista que o participante possivelmente fará o acesso sozinho, entendemos que essa estratégia minimizará a possibilidade de descoberta do padrão utilizado na alocação e as chances de o participante burlar a randomização.

5.4 A INTERVENÇÃO

Imediatamente após a randomização, os participantes do grupo intervenção serão direcionados para o *website* i-HPV e receberão informações sobre o HPV e a vacina HPV. Este *website* será desenvolvido em versão *mobile*, porém, acessível a partir de *desktops* também. O conteúdo será produzido tendo como base teórica a TMP e posteriormente será revisado, apoiado nas melhorias identificadas após a realização de um grupo focal com PVHA e/ou trabalhadores de instituições direcionadas a assistência dessa população chave. Para esse grupo, o *website* será desenvolvido em quatro seções sequenciais, com linguagem simples, acessível e design que segue a tendência de rolagem intuitiva de telas, típicas de *smartphones*. As quatro seções são:

i. Saiba mais sobre o HPV: esta sessão incluiu informações sobre prevalência do HPV, transmissão, doenças relacionadas ao HPV e risco aumentado para PVHA. Nessa seção foram utilizados como construtos teóricos a **gravidade percebida** e a **vulnerabilidade percebida**.

ii. Conheça a vacina HPV: nesta sessão são fornecidas informações sobre a vacina HPV, sua eficácia, recomendações para a população chave e depoimentos com foco voltado para as razões de PVHA se vacinarem. O construto teórico utilizado é a **eficácia da resposta**, neste caso, da vacinação.

iii. Perguntas frequentes: trata-se de uma sessão de perguntas e respostas utilizada para fornecer informações relacionadas às barreiras e preocupações sobre o HPV e a vacina HPV obtidas de pesquisas anteriores e do grupo focal realizado antes da intervenção. Aqui, será explorada a variável de **custo de resposta**.

iv. Vacine-se: esta sessão fornece informações dos locais de vacinação e documentos necessários para obter a vacina. Nesta etapa o construto da **autoeficácia** passa a ser explorado, pois o participante será conduzido a avaliar sua intenção em se vacinar dentro de determinado período de tempo.

5.5 O GRUPO CONTROLE

Os participantes do grupo controle, imediatamente após a randomização, serão direcionados para o *website* MS-HPV. Nele também serão apresentadas informações sobre o HPV e a vacina HPV, porém obtidas do site http://saude.gov.br/saude-de-a-z/hpv dedicado a informar a população em geral sobre o HPV e mantido pelo MS. Para evitar grande disparidade no conteúdo entre os grupos, está será feito um recorte das informações desse site para uma nova página desenvolvida para o projeto. O conteúdo será reproduzido buscando manter a correspondência com os temas abordados na página do grupo intervenção. Dessa forma, serão reproduzidas as seguintes sessões: “O que é o HPV”, “Sinais e Sintomas”, “Prevenção” e “Perguntas e Respostas”. Nesta versão será adicionado o mesmo conteúdo da seção “Vacine-se”, apresentada ao grupo intervenção.

5.6 COLETA DE DADOS

Tanto no iHPV quanto no MS-HPV, assim que o participante iniciar a navegação da seção “Vacine-se”, será indagado sobre sua intenção em se vacinar nos próximos três meses. Sua resposta permitirá capturar o desfecho primário da intervenção. Ela também serve de gatilho para o início da fase de coleta de dados autodeclarados.

Será feita de duas formas: coleta automática via dados de navegação e questionário. Na coleta automática serão contabilizados a origem e tempo de acesso às páginas. Por meio de questionários, serão coletados dados sócio-demográficos, intenção em se vacinar, avaliação da intervenção, as razões para a não vacinação. Todos os dados serão coletados eletronicamente e salvos em banco de dados com acesso restrito aos pesquisadores.

6. PROPOSTA DE ANÁLISE

6.1 VARIÁVEIS

As variáveis e os objetivos que elas responderão estão descritas a seguir.

6.1.1 Viabilidade dos convites

Será medida pela taxa de acessos em cada estratégia de divulgação. A viabilidade dos convites físicos (VCF) será medida da pela razão entre o número de acessos à página inicial P0 originados de pôsteres e panfletos (nP0PP) e o número estimado de atendimentos a PVHA (nA) realizados nos locais de atendimento, durante o período de recrutamento.

= [illegible]

Para viabilidade dos convites *online* (VCO), a situação ideal seria medir a quantidade de acessos a P0 originados de *posts online* (nP0PO) em razão da quantidade de visualização destes mesmos *posts*. Essa situação não é possível já que a divulgação será feita via anúncios gratuitos e em contas não gerenciadas pelo projeto. Assim, consideraremos como denominador o total de seguidores, no último dia de recrutamento, nas mídias sociais das instituições listadas no item 4.1.2 onde os *posts* deverão ser publicados (nS).

= 0 [illegible]

Dessa forma, será possível comparar as estratégias, avaliando o respectivo desempenho no recrutamento dos participantes.

6.1.2 Aceitabilidade

Será expressa pelo percentual de participantes satisfeitos nas três dimensões de análise:

a) Qualidade da informação
b) Qualidade do website
c) Utilidade da intervenção

E pelos indicadores abaixo:

d) Taxa de abandono: Razão entre a quantidade de participantes que entram e saem de nenhuma interação e o total de acessos à página.
e) Tempo médio de permanência, por página e por seção

6.1.3 Eficácia

A eficácia da intervenção (E) será obtida pelo percentual de participantes que declararam intenção em se vacinar. Medido pela razão entre a soma do número de participantes que declaram que irão ou pretendem se vacinar (nV) e o número de participantes dos grupos randomizados para seus respectivos *websites* (nP).

$$= \frac{}{} \times 100$$

6.2 METODOLOGIA DE ANÁLISE DOS DADOS

Será realizada uma análise descritiva da distribuição da frequência das variáveis de desfecho principal e das demais variáveis. As variáveis contínuas serão expressas como média ± desvio padrão com intervalo de confiança (IC) de 95% e as categóricas, em frequências e percentuais. Para a avaliação dos efeitos da intervenção na intenção em se vacinar (eficácia), inicialmente, os grupos serão avaliados quanto à homogeneidade da amostra em relação às características demográficas e relacionadas à saúde. Diferenças entre os grupos estudados serão calculadas por meio do teste T de *Student* para variáveis quantitativas e, para as variáveis categóricas, a comparação entre os grupos será realizada pelo teste do Qui-quadrado. Será utilizado o modelo de regressão logística para comparar os grupos do estudo em todos os desfechos, ajustando-se para as potenciais variáveis de confundimento. As análises estatísticas serão feitas no *software* Stata 12. Valores de $p < 0,05$ serão considerados estatisticamente significativos.

6.2.1 Desfecho primário

Percentual de PVHA com intenção de receber a vacina HPV.

6.2.2 Desfecho secundário

Taxa de iniciação da vacinação (pelo menos uma dose da vacina) entre PVHA e taxa de vacinação completa (três doses da vacina).

6.2.3 Tamanho da amostra

A meta será incluir 836 a 650 participantes (assumindo uma taxa de desistência de 30% a 10%) ou 586 participantes avaliáveis. Obtendo-se uma amostra de 293 pacientes avaliáveis por grupo e adotando-se uma probabilidade de erro tipo I (alfa) de 5%, teremos um poder de 80% de detectar uma diferença de 10% (20% vs. 30%) ou maior entre o grupo intervenção e o grupo controle.

7. CRONOGRAMA

DESCRIÇÃO	
**2021**	Submissão ao CEP (Fevereiro)
	Finalizar protótipo (Abril)
	Discussão com grupo focal (Maio)
	Revisão e ajustes do protótipo após discussão com grupo focal (Maio)
**2022**	Coleta de dados (Janeiro a Junho)
	Análise de dados (Outubro a Novembro)
	Elaboração da tese e artigo (Novembro a Dezembro)

8. ORÇAMENTO

O projeto terá custo relativamente baixo, portanto, será executado com recursos próprios e da taxa de bancada do CNPq do orientador. Serão utilizados equipamentos (computadores, tablets etc.) e software do LEMB para condução da análise dos dados.

**Item**	**Quantidade**	**Total**
Resma de papel	1	57,90
Consultoria de Web designer	40hs	1.800,00
Consultoria de Especialista em Comunicação Social	40hs	1.800,00
**TOTAL**		**R$ 3.657,90**

9. CONSIDERAÇÕES SOBRE A ÉTICA

Serão atendidas todas as Diretrizes e Normas Regulamentadoras de Pesquisa Envolvendo Seres Humanos - Resolução nº466/2012 do Conselho Nacional de Saúde. Os princípios da bioética (autonomia, não-maleficência, beneficência e justiça) serão respeitados (CNS, 2012). Para fins deste estudo, os participantes da pesquisa serão esclarecidos quanto aos objetivos e conteúdo da pesquisa em linguagem acessível em formulário *online*. Portanto, não será possível à aplicação de uma versão assinada de Termo de Consentimento Livre e Esclarecido (TCLE). Serão, ainda, informados de que a participação no estudo é totalmente voluntária e de que o consentimento para participação no estudo pode ser retirado a qualquer momento. Nenhum participante poderá entrar no estudo antes de ter sido obtido o consentimento

O protocolo do estudo e outros documentos relevantes serão submetidos ao Comitê de Ética em Pesquisa para avaliação e aprovação colegiada. O banco de dados será criado sem informações sobre a identidade dos participantes no estudo, portanto todas as análises serão realizadas anonimamente e com garantia da privacidade dos sujeitos incluídos na pesquisa. As informações coletadas serão usadas somente para a finalidade do estudo e os resultados encontrados serão divulgados em periódicos científicos da área.

9.1 AVALIAÇÃO DOS RISCOS E BENEFÍCIOS:

9.1.1 Riscos

Risco de constrangimento com algumas perguntas. Porém, mitigado pelo fato de que o participante poderá fazê-lo com privacidade uma vez que se trata de questionário *online*.

9.1.2 Benefícios

Benefício para o indivíduo que, participando do estudo, poderá optar por receber a vacina. Secundariamente, o indivíduo, estando no posto de vacinação, também tende a atualizar o cartão de vacina, recebendo as doses porventura ainda pendentes. Identificação de uma abordagem mais efetiva na promoção da vacina HPV entre PVHA.

Além disso, o estudo gerará um produto que ficará disponível gratuitamente para uso e adaptações: a intervenção iHPV. Além disso, o trabalho fornece um norte para a produção de informação relevante e eficiente uma vez que a identificará os construtos da TMP e conteúdos relacionados ao HPV e à vacina HPV que mais prenderam a atenção da população chave.

10. REFERÊNCIAS

ABRAMOWITZ, L. *et al.* Human papillomavirus genotype distribution in anal cancer in France: the EDiTH V study. **International Journal of Cancer**, v. 129, n. 2, p. 433–439, 2011.

ABREU, M. N. S. *et al.* Conhecimento e percepção sobre o HPV na população com mais de 18 anos da cidade de Ipatinga, MG, Brasil. **Ciências & Saúde Coletiva**, v. 23, n. 3, p. 849–860, 2018.

ANDRADE, J. *et al.* **Guia de imunização SBIm/SBI – HIV/Aids 2016-2017**. São Paulo: [s.n.]. Disponível em: <https://sbim.org.br/publicacoes/guias/567-guia-de-imunizacao-sbim-sbi-hiv-aids-2016-2017>.

ARBYN, M. *et al.* EUROGIN 2011 roadmap on prevention and treatment of HPV-related disease. **International Journal of Cancer**, v. 131, n. 9, p. 1969–1982, 2012.

BEACHLER, D. C. *et al.* Incidence and risk factors of HPV-related and HPV-unrelated Head and Neck Squamous Cell Carcinoma in HIV-infected individuals. **Oral Oncology**, v. 50, n. 12, p. 1169–1176, 2014.

BLOMBERG, M. *et al.* Genital warts and risk of cancer: A danish study of nearly 50000 patients with genital warts. **Journal of Infectious Diseases**, v. 205, n. 10, p. 1544–1553, 2012.

BOSCH, F. X. *et al.* The causal relation between human papillomavirus and cervical cancer. **Journal of Clinical Pathology**, v. 55, n. 4, p. 244–265, 2002.

BRASIL. Informe técnico da ampliação da oferta das vacinas papilomavírus humano 6, 11, 16 e 18 (recombinante) – vacina HPV quadrivalente e meningocócica C (conjugada). **Ministério da Saúde. Secretaria de Vigilância em Saúde. Departamento De Vigilância das Doenças Transmissíveis. Coordenação-Geral do Programa Nacional de Imunizações.**, v. 18, p. 1–39, 2018.

___. Informe técnico da oferta da vacina papilomavírus humano 6, 11, 16 e 18 (recombinante) – vacina HPV quadrivalente. **Ministério da Saúde. Secretaria de Vigilância em Saúde. Departamento De Vigilância das Doenças Transmissíveis. Coordenação-Geral do Programa Nacional de Imunizações.**, p. 27, 2019.

BRUNI, L. *et al.* **Human Papillomavirus and Related Diseases ReportICO/IARC Information Centre on HPV and Cancer (HPV Information Centre)**. [s.l: s.n.]. Disponível em: <https://www.hpvcentre.net/statistics/reports/XWX.pdf>.

CAMERINI, A. L. *et al.* Using protection motivation theory to predict intention to adhere to official MMR vaccination recommendations in Switzerland. **SSM - Population Health**, v. 7, n. November 2018, p. 100321, 2019.

CDC. **HPV and Cancer**. Disponível em:

<https://www.cdc.gov/cancer/hpv/statistics/index.htm>. Acesso em: 13 mar. 2019.

CETIC.BR. **Tic domicílios. Pesquisa Sobre o Uso das TecnologiasPesquisa sobre o uso das tecnologias de informação e comunicação nos domicílios brasileiros: TIC domicílios 2017**. São Paulo: [s.n.].

CHATURVEDI, A. K. *et al.* Risk of human papillomavirus-associated cancers among persons with AIDS. **Journal of the National Cancer Institute**, v. 101, n. 16, p. 1120–1130, 2009.

COELHO, P. L. S. *et al.* Segurança da vacina papillomavirus humano 6, 11, 16 e 18 (recombinante): revisão sistemática e metanálise. **Revista Paulista de Pediatria**, v. 33, n. 4, p. 474–482, 2015.

DENNY, L. *et al.* Safety and immunogenicity of the HPV-16/18 AS04-adjuvanted vaccine in HIV-positive women in South Africa: A partially-blind randomised placebo-controlled study. **Vaccine**, v. 31, n. 48, p. 5745–5753, 2013.

DENNY, L. A. *et al.* Human papillomavirus, human immunodeficiency virus and immunosuppression. **Vaccine**, v. 30, n. SUPPL.5, p. F168–F174, 2012.

DREYER, G. Clinical implications of the interaction between HPV and HIV infections. **Best Practice and Research: Clinical Obstetrics and Gynaecology**, v. 47, p. 95–106, 2018.

FONTENOT, H. B. *et al.* Increasing HPV vaccination and eliminating barriers: Recommendations from young men who have sex with men. **Vaccine**, v. 34, n. 50, p. 6209–6216, 2016.

FRISCH, M. Human Papillomavirus-Associated Cancers in Patients With Human Immunodeficiency Virus Infection and Acquired Immunodeficiency Syndrome. **Journal of the National Cancer Institute**, v. 92, n. 18, p. 1500–1510, 2000.

GAINFORTH, H. L.; CAO, W.; LATIMER-CHEUNG, A. E. Determinants of human papillomavirus (HPV) vaccination intent among three canadian target groups. **Journal of Cancer Education**, v. 27, n. 4, p. 717–724, 2012.

GAINFORTH, H. L.; LATIMER, A. E. Risky business: Risk information and the moderating effect of message frame and past behaviour on women's perceptions of the Human Papillomavirus vaccine. **Journal of Health Psychology**, v. 17, n. 6, p. 896–906, 2012.

GIACOMET, V. *et al.* Safety and immunogenicity of a quadrivalent human papillomavirus vaccine in HIV-infected and HIV-negative adolescents and young adults. **Vaccine**, v. 32, n. 43, p. 5657–5661, 2014.

HESSOL, N. A. *et al.* Anal intraepithelial neoplasia in a multisite study of HIV-infected and high-risk HIV-uninfected women. **Aids**, v. 23, n. 1, p. 59–70, 2009.

HOULIHAN, C. F. *et al.* HPV infection and increased risk of HIV acquisition . A

systematic review and meta-analysis. **Europe PMC Funders Manuscripts**, v. 26, n. 17, p. 1–18, 2013.

IARC. **World Cancer Day 2019 IARC: “HPV vaccination is safe , effective , and critical for eliminating cervical cancer"**. Disponível em: <https://www.iarc.fr/wp-content/uploads/2019/02/pr264_E.pdf>. Acesso em: 19 fev. 2018.

INCA. **Estimativa 2018: incidência de câncer no Brasil**. Rio de Janeiro: [s.n.]. Disponível em: <https://www.inca.gov.br/sites/ufu.sti.inca.local/files//media/document//estimativa-incidencia-de-cancer-no-brasil-2018.pdf>.

KAHN, J. A. *et al.* Immunogenicity and safety of the human papillomavirus 6, 11, 16, 18 vaccine in HIV-infected young women. **Clinical Infectious Diseases**, v. 57, n. 5, p. 735–744, 2013.

KELLER, M. J. *et al.* Risk of cervical precancer and cancer among HIV-infected women with normal cervical cytology and no evidence of oncogenic HPV infection. **JAMA - Journal of the American Medical Association**, v. 308, n. 4, p. 362–369, 2012.

KOJIC, E. M. *et al.* Immunogenicity and safety of the quadrivalent human papillomavirus vaccine in HIV-1-infected women. **Clinical Infectious Diseases**, v. 59, n. 1, p. 127–135, 2014.

LEONCINI, E. *et al.* Adult height and head and neck cancer: A pooled analysis within the INHANCE Consortium. **Head and Neck**, v. 36, n. 10, p. 1391, 2014.

LEVIN, M. J. *et al.* Safety and immunogenicity of a quadrivalent human papillomavirus (types 6, 11, 16, and 18) vaccine in HIV-infected children 7 to 12 years old. **Journal of Acquired Immune Deficiency Syndromes**, v. 55, n. 2, p. 197–204, 2010.

LEWIS, R. M. *et al.* Prevalence of Genital Human Papillomavirus among Sexually Experienced Males and Females Aged 14-59 Years, United States, 2013-2014. **Journal of Infectious Diseases**, v. 217, n. 6, p. 869–877, 2018.

LIU, R. *et al.* Analysis of hepatitis B vaccination behavior and vaccination willingness among migrant workers from rural China based on protection motivation theory. **Human Vaccines and Immunotherapeutics**, v. 12, n. 5, p. 1155–1163, 2016.

LOOKER, K. J. *et al.* Evidence of synergistic relationships between HIV and Human Papillomavirus (HPV): systematic reviews and meta-analyses of longitudinal studies of HPV acquisition and clearance by HIV status, and of HIV acquisition by HPV status. **Journal of the International AIDS Society**, v. 21, n. 6, 2018.

MACHALEK, D. A. *et al.* Anal human papillomavirus infection and associated neoplastic lesions in men who have sex with men: A systematic review and meta-analysis. **The Lancet Oncology**, v. 13, n. 5, p. 487–500, 2012.

MACHALEK, D. A. *et al.* Very Low Prevalence of Vaccine Human Papillomavirus

Types Among 18- to 35-Year Old Australian Women 9 Years Following Implementation of Vaccination. **The Journal of Infectious diseases**, v. 217, p. 1590–1600, 2018.

MADDUX, J. E.; ROGERS, R. W. Protection motivation and self-efficacy: A revised theory of fear appeals and attitude change. **Journal of Experimental Social Psychology**, v. 19, n. 5, p. 469–479, 1983.

MARTEL, C. DE *et al.* Worldwide burden of cancer attributable to HPV by site, country and HPV type. **International Journal of Cancer**, v. 141, n. 4, p. 664–670, 2017.

MCREE, A. L. *et al.* Outsmart HPV: Acceptability and short-term effects of a web-based HPV vaccination intervention for young adult gay and bisexual men. **Vaccine**, v. 36, n. 52, p. 8158–8164, 2018.

MENDES LOBÃO, W. *et al.* Low coverage of HPV vaccination in the national immunization programme in Brazil: Parental vaccine refusal or barriers in health-service based vaccine delivery? **PloS one**, v. 13, n. 11, p. e0206726, 2018.

MOREIRA, E. D. *et al.* Knowledge and attitudes about human papillomavirus, Pap smears, and cervical cancer among young women in Brazil: Implications for health education and prevention. **International Journal of Gynecological Cancer**, v. 16, n. 2, p. 599–603, 2006.

MOSCICKI, A. B. *et al.* Human papillomavirus infection and abnormal cytology of the anus in HIV-infected and uninfected adolescents. **Aids**, v. 17, n. 3, p. 311–320, 2003.

NOWAK, R. G. *et al.* Increases in human papillomavirus detection during early HIV infection among women in Zimbabwe. **Journal of Infectious Diseases**, v. 203, n. 8, p. 1182–1191, 2011.

NYGÅRD, M. *et al.* Evaluation of the long-term anti-human papillomavirus 6 (HPV6), 11, 16, and 18 immune responses generated by the quadrivalent HPV vaccine. **Clinical and Vaccine Immunology**, v. 22, n. 8, p. 943–948, 2015.

NYITRAY, A. G. The epidemiology of anal human papillomavirus infection among women and men having sex with women. **Sexual Health**, v. 9, n. 6, p. 538–546, 2012.

PEREIRA, R. G. V. *et al.* A influência do conhecimento na atitude frente à vacina contra o Papilomavírus Humano: ensaio clínico randomizado. **ABCS Health Sciences**, v. 41, n. 2, p. 78–83, 2016.

PERNOT, S. *et al.* Immunity and squamous cell carcinoma of the anus: Epidemiological, clinical and therapeutic aspects. **Clinics and Research in Hepatology and Gastroenterology**, v. 38, n. 1, p. 18–23, 2014.

POLLOCK, K. G. J. *et al.* Reduction of low- and high-grade cervical abnormalities associated with high uptake of the HPV bivalent vaccine in Scotland. **British Journal of Cancer**, v. 111, n. 9, p. 1824–1830, 2014.

POP-BRASIL. **Estudo epidemiológico sobre a prevalência nacional de infecção pelo hpv**. [s.l: s.n.].

REITER, P. L. *et al.* Increasing human papillomavirus vaccination among young gay and bisexual men: A randomized pilot trial of the outsmart HPV intervention. **LGBT Health**, v. 5, n. 5, p. 325–329, 2018.

ROGERS, R. W. A Protection Motivation Theory of Fear Appeals and Attitude Change1. **The Journal of Psychology**, v. 91, n. 1, p. 93–114, 1975.

RUBIN, M. A. *et al.* Detection and typing of human papillomavirus DNA in penile carcinoma: Evidence for multiple independent pathways of penile carcinogenesis. **American Journal of Pathology**, v. 159, n. 4, p. 1211–1218, 2001.

SCHILLER, J. T.; CASTELLSAGUÉ, X.; GARLAND, S. M. A review of clinical trials of human papillomavirus prophylactic vaccines. **Vaccine**, v. 30, n. SUPPL.5, p. F123–F138, 2012.

SCHIM VAN DER LOEFF, M. F. *et al.* HPV and anal cancer in HIV-infected individuals: a review. **Current HIV/AIDS reports**, v. 11, n. 3, p. 250–262, 2014.

SCHWARZ, T. F. *et al.* A ten-year study of immunogenicity and safety of the AS04-HPV-16/18 vaccine in adolescent girls aged 10-14 years. **Human Vaccines and Immunotherapeutics**, v. 15, n. 7–8, p. 1970–1979, 2019.

SHVETSOV, Y. B. *et al.* Duration and Clearance of Anal Human Papillomavirus (HPV) Infection among Women: The Hawaii HPV Cohort Study. **Clinical Infectious Diseases**, v. 48, n. 5, p. 536–546, 2009.

SILVA, L. E. L. DA; OLIVEIRA, M. L. C. DE; GALATO, D. Receptividade à vacina contra o papilomavírus humano: uma revisão sistemática. **Rev Panam Salud Publica**, v. 43, p. 1–9, 2019.

SMITH, M. A. *et al.* Fall in Genital Warts Diagnoses in the General and Indigenous Australian Population Following Implementation of a National Human Papillomavirus Vaccination Program : Analysis of Routinely Collected National Hospital Data. **The Journal of Infectious diseases**, v. 211, p. 91–99, 2015.

TOZZI, A. E. *et al.* Comparison of Quality of Internet Pages on Human Papillomavirus Immunization in Italian and in English. **Journal of Adolescent Health**, v. 46, n. 1, p. 83–89, 2010.

VELDHUIJZEN, N. J. *et al.* The epidemiology of human papillomavirus infection in HIV-positive and HIV-negative high-risk women in Kigali, Rwanda. **BMC Infectious Diseases**, v. 11, n. 1, p. 333, 2011.

WHO. **Guide to Introducing HPV Vaccine Into National Immunization Programmes**. Disponível em: <www.who.int/immunization/documents>. Acesso em: 14 mar. 2019.

___. **Weekly epidemiological record**. Disponível em: <https://www.who.int/immunization/policy/position_papers/hpv/en/>. Acesso em: 13 mar. 2019.

___. **Human papillomavirus (HPV) and cervical cancer**. Disponível em: <https://www.who.int/en/news-room/fact-sheets/detail/human-papillomavirus-(hpv)-and-cervical-cancer#>. Acesso em: 13 mar. 2019.

WILKIN, T. *et al.* Safety and Immunogenicity of the Quadrivalent Human Papillomavirus Vaccine in HIV-1–Infected Men. **The Journal of Infectious Diseases**, v. 202, n. 8, p. 1246–1253, 2010.

WILLIAMSON, A.-L. The Interaction between Human Immunodeficiency Virus and Human Papillomaviruses in Heterosexuals in Africa. **Journal of Clinical Medicine**, v. 4, n. 4, p. 579–592, 2015.
