# Supplementary material for: A randomized, controlled trial of a web-based tailored intervention to increase human papillomavirus vaccination among people living with HIV/AIDS
Source: PLoS One. 2025 Mar 31;20(3):e0319646. doi: 10.1371/journal.pone.0319646 (PMC11957270; doi:10.1371/journal.pone.0319646)
Supplement: S2 File — (PDF) [file pone.0319646.s003.pdf]

RESEARCH PROJECT

MOBILE DEVICE INTERVENTION TO INCREASE HPV VACCINE COVERAGE IN PEOPLE LIVING WITH HIV/AIDS

Proposing institution : Gonçalo Moniz Institute – FIOCRUZ. Address: Waldemar Falcão Street, no. 121, Candeal, Salvador-BA. Postal Code: 40.296-710

Team:

Gonçalo Moniz Institute - FIOCRUZ

Dr. Edson Duarte Moreira Junior
Responsible researcher.
Head of the Laboratory of Molecular Epidemiology and Biostatistics (LEMB),
http://lattes.cnpq.br/9530545028476035

Ms. Kaliane Caldas de Brito
PhD student in the postgraduate course in Biotechnology in Health and Investigative Medicine and Health Technologist at the Gonçalo Moniz Institute Email:
Lattes: http://lattes.cnpq.br/6425155220370314

Salvador – Bahia
2020

SUMMARY

INTRODUCTION : Compared to the general population, people living with HIV/AIDS (PLWHA) have a considerably increased risk of all types of anogenital cancers associated with HPV. This virus is responsible for virtually all cases of cervical cancer, anal cancer and genital warts, and for the majority of cases of vaginal, vulvar, penile and oropharyngeal cancers, representing a major public health problem. The HPV vaccine was introduced by the National Immunization Program (PNI) in 2014, but coverage rates are low. Knowledge about the vaccine and lack of/low perception of the risk of infection are highlighted as important factors among those who refuse the vaccine. **O BJECTIVE** : To assess the feasibility, acceptability, and effectiveness of a mobile-based intervention containing information about HPV and the HPV vaccine to promote vaccination among PLWHA. The primary outcome to be assessed is the intention to get vaccinated. **MATERIALS AND METHODS** : An intervention study, a randomized controlled trial, will be conducted with PLWHA, divided into two groups. One of the groups will be presented with information about HPV and the HPV vaccine, developed based on the Protection Motivation Theory (PMT) in a *web intervention* developed for mobile devices. Another group will receive a clipping of information from the page maintained by the Ministry of Health dedicated to informing the population about HPV and the HPV vaccine. Eligible participants are people between 18 and 45 years old, living with HIV, who consented to participate in the study, and who have not received any dose of the HPV vaccine. The intention to get vaccinated will be obtained by the percentage of participants who declare that they will or intend to get vaccinated in the next three months. The quality of the intervention will also be assessed by the research participants.

Keywords: Human Papillomavirus; HPV Vaccine; Human Immunodeficiency Virus

SUMMARY

1. INTRODUCTION 5

2. HYPOTHESIS 7

3. LITERATURE REVIEW 8

3.1 THE ASSOCIATION BETWEEN HPV AND ANOGENITAL AND HEAD AND NECK MALIGNANITIES 8

3.1.1 Cervical cancer 8

3.1.2 Other anogenital cancers: anus, vulva, vagina, penis....................................... 9

3.1.3 Head and neck cancers 9

3.2 HPV-ASSOCIATED DISEASES IN PEOPLE LIVING WITH HIV/AIDS 11

3.3.1 Cervical cancer 11

3.3.2 Anal cancer... 12

3.3.3 Vulvar/vaginal cancer 13

3.3.4 Penile cancer 13

3.3.5 Head and neck cancer.. 14

3.3 THE HPV VACCINE 15

1. INTRODUCTION

Human papillomavirus (HPV) is a virus that infects the male and female anogenital epithelium and is the most common viral infection of the reproductive tract. Most sexually active individuals will acquire HPV infection at some point in their lives (WHO, 2019) . The causal association between HPV and cervical cancer is well established, and there is growing evidence that HPV is a relevant factor in other anogenital cancers (anus, vulva, vagina, and penis), as well as in head and neck cancers (ARBYN *et al.* , 2012; BOSCH *et al.* , 2002; BRUNI *et al.* , 2019; SCHIM VAN DER LOEFF *et al.* , 2014) .

Observational studies suggest that coinfections with the human immunodeficiency virus (HIV) and HPV may have multiple interactions (LOOKER *et al.* , 2018) . The interaction between the two sexually transmitted infections (STIs) appears to be related to altered immunity, increased susceptibility, and possibly reactivation of latent HPV infection (DENNY *et al.* , 2012) . Women living with HIV/AIDS have a 5-fold increased risk of developing cervical cancer and almost 6-fold increased risk of developing vulvar/vaginal cancer when compared to women not infected with HIV. Among men, the chance of developing anal cancer increases by 38-fold (FRISCH, 2000) . Anal HPV infections are almost universal among HIV-infected men who have sex with men (MSM) (SCHIM VAN DER LOEFF *et al.* , 2014) .

There are more than 100 types of HPV, of which at least 14 are cancer-causing. High-risk oncogenic HPV types are detected in approximately 90% of anal cancer cases and virtually 100% of cervical cancers, the most common being HPV-16 and HPV-18 types, which together are responsible for approximately 70% of all cervical cancer cases worldwide (ABREU *et al.* , 2018; ARBYN *et al.* , 2012; BRUNI *et al.* , 2019) . Non-carcinogenic HPV types (especially HPV-6 and HPV-11 types) are associated with up to 90% of anogenital warts (BRASIL, 2018; CDC, 2019) .

Currently, we have three vaccines that are considered very effective and safe for preventing lesions caused by HPV: the quadrivalent vaccine, the bivalent vaccine and the nonavalent vaccine, which differ basically in terms of the types of HPV against which they provide protection and are recommended by the WHO (WHO, 2017a) . In Brazil, the Ministry of Health (MS) has made the quadrivalent HPV vaccine available ,

which offers protection against the 4 types (6, 11, 16 and 18) of HPV since 2014, when it was incorporated into the Unified Health System (SUS). Initially restricted to girls, as of March 2015 it also included the female population aged 9 to 26 years old living with HIV/AIDS. Two years later, in 2017, the target population was expanded again and currently includes girls aged 9 to 14, boys aged 11 to 14, women and men aged 9 to 26 living with HIV/AIDS, and individuals who have undergone solid organ and bone marrow transplants, as well as cancer patients. For both sexes, the goal is to vaccinate 80% of the target population, administered in 2 doses with a minimum interval of 6 months between them. In the case of PLWHA, the vaccination schedule is different, with 3 doses administered, with the second dose administered after 2 months and the third after 6 months of the initial dose (BRASIL, 2018) .

Although immunization is an important component in the prevention of opportunistic infections among PLWHA, vaccination coverage, especially among adults, is low and its decline has been observed in several countries, including Brazil. In 2018, the cumulative vaccination coverage of the HPV vaccine among girls was 70.3% and 49.9% for the first and second doses, respectively. Among boys, vaccination coverage of the first dose was 42.3% and 20.1% for the second dose (BRASIL, 2019a) . Doubts regarding the safety and efficacy of vaccines are among the main reasons for not updating the vaccination schedule among this group. However, in general, vaccination against HPV is over 95% effective in preventing cervical abnormalities and genital warts (ANDRADE *et al.* , 2016) .

The introduction of the HPV vaccine into National Immunization Programs is considered complicated by several factors, including the need for interlocutors to reach such a diverse target population, the sensitivity surrounding a vaccine that protects against an STI, and the need for robust communication and social mobilization to help with acceptance, ensure completion of the plan, and reduce costs related to tracking cases of abandonment (WHO, 2016) . The difficulty in creating effective messages is highlighted as an important challenge to be overcome in promoting the HPV vaccine:

> Accurate, technical, culturally appropriate, practical and motivational information should be conveyed in a way that is easily understood by different audiences at different times (WHO, 2017b, P.50)

2. HYPOTHESIS

With the purpose of helping to create communication strategies that launch a call to action, this work has the following **guiding question: To what extent is an intervention, based on mobile devices, to promote HPV vaccination among young adults living with HIV/AIDS more efficient than traditional, *web -based interventions* currently used by the Ministry of Health to inform about HPV and the HPV vaccine for PLWHA?**

The hypothesis is that information about HPV and the HPV vaccine, when presented concisely, in direct language, with more visual resources, less text and with content developed based on the theoretical basis of the Protection Motivation Theory (PMT), is more efficient in arousing the intention to get vaccinated. Therefore, this study aims to evaluate the feasibility, acceptability and effectiveness of a *website intervention* (aimed at mobile devices) containing information about HPV and the HPV vaccine to promote vaccination among people living with HIV/AIDS **.** In this way, it is expected to contribute to the improvement of outreach actions to promote the HPV vaccine among this key population.

3. LITERATURE REVIEW

3.1 THE ASSOCIATION BETWEEN HPV AND ANOGENITAL AND HEAD AND NECK MALIGNANITIES

HPV is a virus that infects the male and female anogenital epithelium and is the most common viral infection of the reproductive tract (WHO, 2019) . It forms a highly diverse group of more than 100 types, which are usually grouped by their oncogenic potential. Regarding this potential, they can be classified as low risk (types 6, 11, 42, 43 and 44) or high risk (types 16, 18, 31, 33, 35, 39, 45, 46, 51, 52, 56,58, 59 and 68) (ABREU *et al.* , 2018; ARBYN *et al.* , 2012) . HPV infection causes, globally, more than 600 thousand cases of cervical, vulvar, vaginal, penile, anal and oropharyngeal cancer each year, in addition to benign diseases, such as genital warts (MARTEL, de *et al.* , 2017) .

3.1.1 Cervical cancer

HPV is a necessary cause of cervical cancer (BOSCH *et al.* , 2002) . HPV-16 and HPV-18 types are responsible for approximately 70% of all cervical cancer cases worldwide, with HPV-16 alone causing approximately 50% of all cases (ABREU et al., 2018). Cervical cancer is a major public health problem due to its high incidence and mortality. It is the third most common cancer among women, the second among women aged 15 to 44. Globally, an estimated 569,847 new cases and 311,365 deaths from cervical cancer were recorded in 2018 (BRUNI et al., 2019). More than 85% of these deaths occur in low- and middle-income countries, especially in sub-Saharan African countries, which have the highest HIV rates (WHO, 2019) . In Brazil, cervical cancer is the third leading cause of cancer death among women and claims 5,000 lives each year (BRASIL, 2018) . More than 16,000 new cases are estimated for each year of the 2018-2019 biennium, with an estimated risk of 15.43 cases per 100,000 women (INCA, 2017) . Projections from the International Agency for Research on Cancer show that, if preventive measures are not taken and implemented quickly, by 2040, the number of deaths caused by cervical cancer will increase by 50% compared to 2018 (IARC, 2019) .

3.1.2 Other anogenital cancers: anus, vulva, vagina, penis

Globally, each year, approximately 8,500 cases of vulvar carcinoma, 12,000 of vaginal cancer, 35,000 of anal cancer and 13,000 of penile cancer are attributed to HPV (MARTEL, de *et al.* , 2017) .

Anal cancer is similar to cervical cancer in terms of overall HPV DNA positivity (Bruni *et al.* , 2019) . HPV-16 and HPV-18 types account for 87% of all cases (MARTEL, de *et al.* , 2017) , with HPV-16 being the most prevalent (75%) (ABRAMOWITZ *et al.* , 2011) . Almost 90% of anal cancers are attributable to HPV, and globally, malignancy is equally distributed between the two sexes (MARTEL, de *et al.* , 2017) . Rare in the general population, with an average worldwide incidence of 1 per 100,000 people (Bruni *et al.* , 2019) , anal cancer occurs slightly more frequently in men in less developed countries and in women in more developed countries (MARTEL, de *et al.* , 2017) . It has also been increasing among populations of men who are MSM, women with a history of cervical or vulvar cancer, and immunocompromised populations, including those infected with HIV (BRUNI *et al.* , 2019; SCHIM VAN DER LOEFF *et al.* , 2014) .

Vulvar cancer is relatively rare among women worldwide, with an estimated 27,000 new cases in 2008. Worldwide, approximately 60% of all vulvar cancer cases occur in more developed countries (BRUNI *et al.* , 2019) . HPV-16 and HPV-18 are responsible for approximately 72% of cases, with 85% of these attributed to HPV-16 alone (MARTEL, de *et al.* , 2017) .

The incidence of vaginal cancer is estimated at 13,000 new cases in 2008 (BRUNI *et al.* , 2019) , of which 78% of cases are attributable to HPV-16 and HPV18 (MARTEL, de *et al.* , 2017) .

Globally, penile cancer was estimated at 22,000 in 2008 (Bruni *et al.* , 2019) . HPV-16 and HPV-18 are attributed to 70% of cases (MARTEL, *et al.* , 2017) . In invasive tumors of the penis, HPV-16 was the most common type detected (40%) (RUBIN *et al.* , 2001) .

3.1.3 Head and neck cancers

Three sites of head and neck cancer have been associated with HPV: oropharynx and, to a much weaker degree, oral cavity and larynx (MARTEL, de *et*

al. , 2017) . A meta-analysis showed that the overall prevalence of HPV in head and neck tumors increased significantly over time: it went from 40.5% before 2000, to 64.3% between 2000 and 2004 and 72.2% between 2005 and 2009 (LEONCINI *et al.* , 2014) .

Globally, approximately 38,000 cases of head and neck cancer are attributable to HPV. Of these, approximately 29,000 are oropharyngeal cancers (which mainly include the tonsils and base of the tongue), 4,400 are oral cavity cancers, and 3,800 are laryngeal cancers (MARTEL, de *et al.* , 2017) . HPV-16 is thought to be responsible for 96% of oropharyngeal cancer cases. Current evidence suggests that HPV-16 and HPV-18 types are also associated with 85% of head and neck cancers (MARTEL, de *et al.* , 2017; WHO, 2017a) .

Table 1 shows the main types of cancer related to HPV, as well as the percentages attributable to HPV-6, HPV-11, HPV-16 and HPV-18, present in the quadrivalent vaccine.

Table 1 – Attributable percentages of cancers related to HPV-16/18 and HPV-6/11/16/18/31/33/45/52/58. Source: Adapted from MARTEL, *et al.* , 2017

**HPV-related cancers**	**HPV attributable number**	**Relative contribution of HPV-16/18**		**Relative contribution of HPV-6/11/16/18/31/33/45/52/58**	
		**Percentage**	**Number**	**Percentage**	**Number**
Cervical	530,000	70	370,000	89	470,000
Anus	35,000	86	30,000	94	33,000
Vulva	8,500	73	6.200	87	7,400
Vagina	12,000	62	7,400	83	9,900
Penis	13,000	70	9.100	85	11,000
Head and Neck	38,000	84	32,000	89	34,000

In addition to the cancer cases described above, HPV is associated with anogenital warts, with HPV-6 and HPV-11 being associated with up to 90% of cases (WHO, 2019) . Furthermore, individuals with genital warts have an increased long-term risk of developing anogenital and head and neck cancers and these risks remained elevated for more than 10 years after the diagnosis of genital warts (BLOMBERG *et al.* , 2012) .

3.2 HPV-ASSOCIATED DISEASES IN PEOPLE LIVING WITH HIV/AIDS

Compared to the general population, HIV-infected individuals have a considerably increased risk for all types of HPV-associated anogenital cancers (FRISCH, 2000) . Current evidence suggests that HIV and HPV infections may interact in several ways. In addition to infecting anogenital sites, they are influenced by similar risk factors, such as the number of sexual partners. HIV-mediated immune impairment causes increased acquisition and persistence of HPV (DREYER, 2018) .

A meta-analysis indicated that the risk of HPV acquisition is approximately doubled in the presence of HIV infection. It also indicated a near doubling of HIV acquisition in those individuals with HPV infection (LOOKER *et al.* , 2018) . A study among women in Zimbabwe found that within 6 months after an incident of HIV infection, anogenital HPV DNA positivity increased markedly - even without an increase in the risky sexual behavior required for infection (NOWAK *et al.* , 2011) . Thus, acquisition of HIV and HPV is increased by existing infection with the other virus, and the clinical course of HPV-related disease is negatively influenced by low immunity (DREYER, 2018) .

People infected with HPV have a higher risk of acquiring HIV than people who are not infected with HPV, even after adjusting for risk factors (HOULIHAN *et al.* , 2013) . In areas of high HIV prevalence, HIV-positive women may cause increases in the prevalence of genital HPV infection in HIV-negative men and thus increase HPV circulating in the community (WILLIAMSON, 2015) . On the other hand, several cross-sectional and longitudinal studies have shown that HIV viral suppression and higher CD4 T cell counts help reduce HPV replication, persistence, and multiple infections (KELLER *et al.* , 2012) .

3.2.1 Cervical cancer

HPV-related malignancies, especially cervical cancer, cause significant morbidity and mortality among HIV-infected groups (DREYER, 2018) . In a study conducted in Zimbabwean women, HIV infection was associated with HPV clearance, indicating that the immune response to HPV may result in an increase in HIV-susceptible cells in the genital tract . Thus, HIV-positive women have a higher

incidence and faster progression of cervical cancer (WILLIAMSON, 2015) . Due to immunosuppression , in addition to having higher rates of HPV and cervical cancer, they are also resistant to treatment for HPV-related diseases and prone to accelerated development of HPV-associated cancer (CHATURVEDI et al., 2009) . HIV is believed to exacerbate the burden of cervical cancer (LOOKER et al., 2018) .

In Rwanda, a study conducted among women at high risk of STIs showed that the prevalence of any HPV was 47% among HIV-negative women and 72% in HIV-positive women (VELDHUIJZEN et al., 2011) . The interval between acquisition of HPV infection and progression to invasive carcinoma is generally 20 years or more (DENNY et al., 2012) . However, in women with untreated HIV infection, this time is reduced to 5 to 10 years (WHO, 2019). A study that followed more than 50,000 women living with HIV/AIDS in the United States for 5 years demonstrated that invasive cervical cancer occurred in statistically significant excess among women living with HIV/AIDS (RR 5.4; 95% CI 3.9-7.2) (FRISCH, 2000).

3.2.2 Anal cancer

Anal infections with high-risk HPV are very common among HIV-positive individuals (ABRAMOWITZ et al., 2011) , especially among MSM (SCHIM VAN DER LOEFF et al., 2014) . This is what the study by Frisch (2000) points out , which also included more than 250,000 men living with HIV/AIDS and demonstrated that invasive and in situ anal cancers occurred in excess, especially among patients under 30 years of age, among whom the RRs were above 100. Among older men, the risk increased significantly by 20 to 40 times. Among women, the RRs for invasive anal cancer (RR 6.8; 95% CI 2.7-14.0) and in situ anal cancer (RR 7.8; 95% CI 0.2-43.6) were as high as for cervical and vulvar/vaginal cancer. Although elevated in all ethnic groups, RRs for anal cancer were higher in whites than in blacks or Hispanics.

A systematic review summarized data suggesting that also among HIV-infected heterosexual men, the prevalence of anal HPV was higher than among HIV-negative men (NYITRAY, 2012) . Other studies have also shown that the prevalence of anal HPV was higher among HIV-infected women than among HIV-negative women. (SCHIM VAN DER LOEFF et al., 2014) .

A prospective study of women over 18 years of age treated at three HIV clinical centers found that low-grade anal intraepithelial neoplasia was present in 12%

of HIV-infected patients and in 5% of HIV-uninfected women. High-grade anal intraepithelial neoplasia was present in 9% of HIV-infected women and in 1% of HIV-uninfected women (HESSOL et al., 2009) . Among adolescents aged 13 to 18 years, girls also had a higher prevalence. While the prevalence of anal HPV infection in HIV-infected boys was 48% versus 36% among uninfected boys, in girls the number was 59% among HIV-seropositive women and 13% among uninfected girls (MOSCICKI et al., 2003) .

The prevalence of anal HPV has rarely been measured among healthy adult women who are HIV-negative (NYITRAY, 2012) . However, a cohort study conducted in Hawaii with 1378 women showed that 50% of the participants had anal HPV infections (SHVETSOV et al., 2009). In a meta-analysis including 8 studies of HIV-negative MSM, the overall prevalence of HPV was 37.2% (MACHALEK et al., 2012) .

3.2.3 Vulvar/vaginal cancer

Women living with HIV/AIDS have a risk of developing vulvar/vaginal cancer almost 6 times higher when compared to women not infected with HIV. In the age group of 0 to 29 years, the RR was 37.2 (95% CI 7.7 to 108.8) for invasive lesions and 14.5 (95% CI 4.0 to 37.1) for in situ lesions (FRISCH, 2000). Although (CHATURVEDI et al., 2009) identified a lower risk for the development of vaginal or vulvar cancer in situ in PLWHA (RR = 1.52, 95% CI = 0.99 to 2.35, P = 0.055)

3.2.4 Penile cancer

In situ penile cancer occurs in excess among men living with HIV/AIDS (RR 6.9; 95% CI 4.2–10.6), particularly among those under 30 years of age (RR 16.1; 95% CI 4.4–41.2). Considering all age groups together, the risks of invasive and *in situ* penile cancer increased fivefold or more among blacks and Hispanics (FRISCH, 2000). Table 2 below shows the relative risks by age of HPV-associated anogenital cancers among the 309,336 patients with HIV/AIDS who participated in the study.

Table 2 – Relative risks by age of HPV-associated anogenital cancers among 309,336 patients with HIV/AIDS. Source: Adapted from FRISCH, 2000

	Relative Risk (95% confidence interval)				
Age	Cervical	Vulva/Vagina	Anus (Women)	Anus (Men)	Penis
			**Invasive Cancer**		
<30	6.1 (2.6–12.0)	37.2 (7.7–108.8)	134.3 (16.3–484.8)	162.7 (103.1–244.0)	37.2 (7.7–108.6)
30-39	5.9 (3.8–8.7)	8.5 (3.1–18.4)	12.2 (2.5–35.7)	40.1 (31.2–50.8)	7.4 (2.4–17.3)
40-49	6.0 (3.1–10.4)	3.0 (0.4–10.8)	2.8 (0.1–15.6)	39.3 (31.3–48.7)	2.2 (0.4–6.3)
>50	—†	1.7 (0.0–9.3)	2.4 (0.1–13.5)	23.4 (16.6–32.0)	1.8 (0.4–5.3)
All	5.4 (3.9–7.2)	5.8 (3.0–10.2)	6.8 (2.7–14.0)	37.9 (33.0–43.4)	3.7 (2.0–6.2)
			**Cancer in *Situ***		
<30	5.3 (4.6–6.1)	14.5 (4.0–37.1)	—	130.4 (71.2–219.0)	16.1 (4.4–41.2)
30-39	4.3 (3.9–4.8)	3.4 (0.9–8.7)	21.0 (0.5–117.1)	72.7 (55.5–93.6)	8.4 (3.6–16.5)
40-49	4.7 (3.9–5.7)	1.0 (0.0–5.8)	—	38.0 (24.3–56.2)	4.8 (1.3–12.4)
>50	4.1 (2.4–6.6)	5.1 (0.6–18.5)	—	40.2 (17.3–79.3)	4.6 (1.3–11.8)
All	4.6 (4.3–5.0)	3.9 (2.0–7.0)	7.8 (0.2–43.6)	60.1 (49.2–72.7)	6.9 (4.2–10.6)

v

3.2.5 Head and neck cancer

In the United States, there is an excess of approximately 50% in oral cavity cancer cases among PLWHA compared to the population. This excess increases to almost 70% among those living with AIDS for five or more years (BEACHLER *et al.* , 2014) .

Warts are caused by low-risk HPV and are more prevalent among HIV-infected people, but their size may not correlate well with immunity (PERNOT *et al.* , 2014) .

In the general population, HPV infection is considerably higher in young people and decreases with age (LEWIS *et al* ., 2018) . Preliminary data from the POP-Brasil project – Epidemiological Study on the National Prevalence of HPV Infection showed that the estimated prevalence of HPV among young people aged 16 to 25 is 54.6% (POP-BRASIL, 2017) . The prevalence profile for HPV-16 is 53.2% and 15.8% for HPV-18 (BRASIL, 2018) .

According to data obtained from the Health Surveillance Secretariat, in 2018, 43,941 new cases of HIV and 37,161 cases of AIDS were diagnosed in Brazil.

Between 2008 and 2018, there was an increase in the detection rate among young people aged 15 to 19 and 20 to 24 years old of 62.2% and 94.6%, respectively. This group alone is estimated to have more than 115,000 people, considering the year of diagnosis between 2008 and 2018. Among women, there was a decrease in the detection rate in all age groups during the same period (BRASIL, 2019b). Compulsory notification of HIV infection began in 2014, which, added to the underreporting factor, makes it difficult to conduct a more rigorous epidemiological analysis of infection trends in Brazil.

3.3 THE HPV VACCINE

The first HPV vaccine (vHPV) was licensed in 2006 and many countries have since included it in their national immunization programs (WHO, 2016) . There are currently three prophylactic HPV vaccines on the market:

i. The quadrivalent vaccine Gardasil (Merck Inc.): first licensed in 2006 and targets oncogenic HPV-16/18 as well as low-risk HPV-6/11 types that cause genital warts.
ii. The bivalent vaccine Cervarix (GlaxoSmithKline): licensed in 2007, offers protection against HPV-16/18.
iii. The nonavalent vaccine Gardasil 9 (Merck Inc.): licensed in 2014, provides protection against HPV-6/11/16/18 and five other more carcinogenic types (HPV-31/33/45/52/58) (MARTEL, *et al.* , 2017)

None of the vaccines contain live biological products or viral DNA and are therefore non-infectious; they do not contain antibiotics or preservatives. In addition to the viral types for which they are intended, they also differ in terms of indication and dosage. The vaccine is not recommended during pregnancy (WHO, 2017) .

After a 3-dose schedule, the quadrivalent and bivalent vaccines were evaluated and shown to be highly immunogenic, with the highest immune responses observed in girls aged 9 to 15 years (SCHILLER, CASTELLSAGUÉ and GARLAND, 2012) . Both confer protection for approximately 10 years (SCHWARZ *et al.* , 2019) . Evaluated in three Phase II/III studies, the quadrivalent vaccine showed seropositivity rates of 97.6%, 96.3%, 100% and 91.4% for HPV-6/11/16/18 types, respectively. (NYGÅRD *et al.* , 2015) . Regarding adverse effects, a meta-analysis that included 14

studies showed that the main adverse effects related to vaccination were pain, erythema, edema and fever (COELHO *et al.* , 2015) .

1.1.1. Efficacy, safety and immunogenicity of the HPV vaccine in PLWHA

A study of quadrivalent vHPV in HIV-infected adolescents and young adults aged 13 to 27 years showed that the vaccine is safe and well tolerated, with a seroconversion rate of 85% in HIV-infected individuals and 91% in HIV-negative individuals. No serious or life-threatening adverse events were reported. The most common local side effect was pain, which occurred in 18.8% of HIV-negative patients and in 32.6% of HIV-infected patients. The most common systemic side effect was headache, reported in 13.5% of HIV-infected individuals and in 2.2% of HIV-negative individuals (GIACOMET et al., 2014) .

The safety and immunogenicity of bivalent vcHPV in HIV-infected women in South Africa were also evaluated. vcHPV was found to be safe in asymptomatic HIV-positive women aged 18 to 25 years and no impacts on CD4 T-cell count, HIV viral load or HIV clinical stage were identified (DENNY et al., 2013).

A study of 319 HIV-infected women in the United States, Brazil, and South Africa showed that quadrivalent vcHPV is safe and immunogenic in HIV-infected women aged 13 to 45 years. The seroconversion rates for the four HPV types (6, 11, 16, and 18) in women with CD4 T-cell counts greater than 350 cells/µL were 96%, 98%, 99%, and 91%, respectively, while women with CD4 counts of <200 cells/µL had conversion rates of 84%, 92%, 93%, and 75%, respectively (KOJIC et al., 2014) . In a clinical trial of HIV-positive MSM vaccinated with three doses of quadrivalent vcHPV , seroconversion rates for all HPV types (6, 11, 16, and 18) were 98%, 99%, 100%, and 95%, respectively. No adverse effects on CD4+ counts or plasma HIV-1 RNA were observed. There were also no grade 3 or higher adverse events attributable to vaccination among the 109 men who received at least 1 dose of the vaccine (Wilkin et al., 2010) .

In HIV-infected children and adolescents, data on the immunogenicity and safety of quadrivalent vcHPV are scarce. To date, few studies have been conducted. However, in a study of a cohort of HIV-infected children and adolescents aged 8 to 11 years, seroconversion for all 4 antigens was 96% higher among those who received quadrivalent vcHPV . In addition to being immunogenic, the vaccine was also shown to be safe (LEVIN et al., 2010) .

Results from a multicenter study of 99 HIV-positive women aged 16 to 23 years indicated that immune responses to quadrivalent vHPV were robust and the vaccine was well tolerated. Seroconversion rates were 100% for HPV-6/11/16/18 among participants on antiretroviral therapy . Rates ranged from 92.3% (for HPV-18) to 100.0% (for HPV-6) among participants not on antiretroviral therapy . One serious adverse event (fatigue) was observed (Kahn et al., 2013) .

Australia, where the quadrivalent vaccine was implemented in the 2007 National Immunization Program and whose coverage rate was over 70% for the three doses in the target population, is now reaping the fruits of its initiative: a 92% reduction in the prevalence of HPV types 16 and 18 among women aged 18 to 35 (MACHALEK et al., 2018) ; a 90% reduction in genital warts in women aged 12 to 17 and 73% in women aged 18 to 26, in addition to a 38% reduction in men aged 18 to 26, indirect protection from vaccination exclusively for women (SMITH et al., 2015) . In Scotland, a significant reduction in diagnoses of cervical intraepithelial neoplasia was also observed in women who received three doses of the vaccine compared to unvaccinated women (POLLOCK et al., 2014) .

Although the literature indicates the high potential of the vaccine in preventing cervical neoplasia and genital lesions, vcHPV has generated controversies that have an impact on acceptance and adherence by users (SILVA, OLIVEIRA and GALATO, 2019) .

In Brazil, a study conducted in 2002 assessed the knowledge and attitudes about HPV, cervical cancer and the Pap smear in 204 women aged 16-23 years, treated at a public hospital. The data revealed that 67% of the participants did not know that HPV can cause cervical cancer/warts and only 10% recognized that HPV could lead to cervical cancer (MOREIRA et al., 2006) .

Mendes Lobão and collaborators (2018) conducted a study to assess the acceptance of the HPV vaccine in Brazilian urban centers after its inclusion in the PNI. For daughters and sons up to 18 years of age, parental acceptance of the HPV vaccine was 92% and 86%, respectively. The study also revealed that those who refused vaccination were less likely to know that HPV is sexually transmitted and causes genital warts. In addition, prevention of cervical cancer and genital warts was less frequently cited by the parents surveyed as a reason for accepting the HPV vaccine. Although acceptance is high, there is no perception of the severity of the diseases for which the vaccine offers protection.

In a systematic review carried out with the aim of characterizing receptivity to the HPV vaccine and describing the barriers and facilitators of this receptivity, 11 facilitators and nine barriers to receptivity were identified. Knowledge about the vaccine stood out among the facilitators and the lack/inadequate information about the vaccine and the absence/low perception of the risk of infection were the most cited barriers (SILVA, OLIVEIRA and GALATO, 2019) .

3.4 THE THEORY OF MOTIVATION FOR PROTECTION

The Protection Motivation Theory (PMT) formed the theoretical basis of the intervention. According to her, when faced with information about a health threat, the motivation to protect oneself arises from the cognitive assessment of the severity of this threat (perceived severity), the perception of the probability of occurrence (perceived vulnerability), together with the belief that a recommended coping response can effectively prevent its occurrence (response efficacy) (Rogers, 1975) . According to Maddux; Rogers (1983) , an important variable that can affect the acceptance of a recommended coping response is the amount of work involved in its implementation (response cost). If an event is not assessed as serious, as likely, or as if nothing can be done about it, then no protective motivation would be aroused and, therefore, there would be no change in behavioral intentions (Rogers, 1975) .

Initially, the TMP postulated that the motivation for protection – and therefore the change in attitude – was a multiplicative function of these three mediating processes. Later, after its revision, the multiplicative notion was abandoned and the cognitive process of self-efficacy was included, that is, the belief that the person has the capacity to successfully execute the response to face the threat. Thus, the perceived severity, vulnerability Perceived and response effectiveness, in interaction with self-efficacy, determine intentions (protective motivation) to engage in a given behavior (MADDUX; ROGERS, 1983) . TMP has been used in several studies related to motivation for health protection (CAMERINI *et al.* , 2019; GAINFORTH, CAO and LATIMER-CHEUNG, 2012; GAINFORTH and LATIMER, 2012; MCREE *et al.* , 2018)

A study conducted in Switzerland with 554 parents of high school students aged 13 to 15 years used the principles of the PMT to identify predictors of parental intention to follow official MMR vaccination recommendations. Response efficacy

(vaccination) was shown to be directly related to parental intention to adhere to MMR vaccination recommendations (CAMERINI *et al.* , 2019) . The PMT was also used to predict hepatitis B vaccination intention and behavior in a population of adult immigrants in China. Perceived vulnerability and response efficacy were significant factors that determined hepatitis B vaccine acceptance (LIU *et al.* , 2016).

A study examined the theoretical determinants of HPV vaccination intentions by assessing the PMT and theory of planned behavior (TPB) constructs in three groups: college women, parents of daughters, and parents of sons. The results indicate that the PMT and TBP constructs predict intentions for the different groups. Focusing on vaccine response efficacy, rather than perceived HPV severity, was most effective in increasing vaccination intentions among all groups. Focusing on HPV vulnerability was effective only among college women and parents of sons, and self-efficacy predicted intentions among college women and parents of daughters (GAINFORTH, CAO, and LATIMER-CHEUNG, 2012) .

A pilot study to test an HPV vaccination intervention among 150 young gay and bisexual men aged 18 to 25 showed that HPV vaccination initiation was higher among those who received HPV information based on the PMT constructs (45% vs. 26%). This same group also showed a higher tendency to complete the full vaccination schedule (11% vs. 3%) (REITER *et al.* , 2018) .

3.5 THE USE OF MOBILE DEVICES FOR HEALTH INFORMATION

According to the 2017 TIC Households survey, which measures the availability and use of Information and Communication Technologies (ICT) in Brazil and is conducted by the Regional Center for Studies for the Development of the Information Society (Cetic.br), the number of Internet users in Brazil reached 120.7 million, representing 67% of the population aged ten or over. Of these, almost all (96%) used the Internet via cell phone, with 49% of them using the network only via this device (CETIC.BR, 2018) . The profile of exclusive use via cell phone was more common among users in classes D and E. and rural areas, reflecting a reality in which low-income citizens do not have multiple devices to access the Internet, as is the case in classes A and B. The survey also showed that 77% of Internet users access social

networks and 44% searched for information related to health or health services on the network in 2017.

Tozzi and colleagues (2010) used search engines to compare the quality of information about HPV and the HPV vaccine on *web pages* in both English and Italian. Scores in the domains of credibility, content, and design were higher on pages from public health agencies or universities. Some of the WHO-accredited websites in both Italy and the United States that include information about HPV immunization were not displayed by the search engines used in the study, suggesting that web pages *with* high-quality information about the HPV vaccine may not be easily retrieved by ordinary users. Most of the *web pages* displayed in this study were privately owned or news-provided websites, which typically host paid advertising or are for-profit.

A study carried out with MSM identified, through a focus group, that the increasing use of mobile strategies was identified as a facilitator for access to health information made available *online* (FONTENOT *et al.* , 2016) . Following this trend, American researchers carried out an intervention with MSM aged between 18 and 26 years old focusing on the use of mobile devices and found that, compared to participants in the control group, intervention participants reported a greater perception that MSM have a higher risk of anal cancer compared to other men, greater self-efficacy in vaccination against HPV and less perceived harm from using the vaccine (MCREE *et al.* , 2018) . At a time when misinformation circulates in large volumes in digital media, it is of utmost importance to have all the means to provide the population with useful and quality information in order to better guide decisions regarding the acceptance of vaccines.

An intervention study conducted to assess prior knowledge and the role of educational action on attitudes toward HPV vaccination in 200 women aged 18 to 30 years revealed that attitudes toward the HPV vaccine were different in the group that received the educational action. This group showed greater unconditional adherence to the vaccine, while the group without intervention conditioned acceptance of the vaccine to obtaining more information or to the recommendation of their attending physician. The researchers identified that only 28.6% of the participants had correct information about the preventive action of the HPV vaccine, and the majority (71.4%) believed that the vaccine had therapeutic purposes (PEREIRA *et al.* , 2016) .

Educational actions combined with mobile technologies and constructs that adequately motivate individuals to seek protection against health-related problems can increase the chances of success of vaccination promotion interventions.

4. OBJECTIVES

4.1 GENERAL OBJECTIVE:

To assess the feasibility, acceptability and effectiveness of a mobile-based intervention containing information about HPV and the HPV vaccine to promote vaccination in people living with HIV/AIDS.

4.2 SPECIFIC OBJECTIVES

4.2.1 Evaluate the effectiveness of the intervention by estimating:

4.2.1.1 Percentage of individuals intending to get vaccinated

4.2.1.2 Percentage of individuals who started vaccination

4.2.2 Determine the acceptability of the intervention with regard to:

4.2.2.1 Quality of information

Website quality

4.2.2.3 Usefulness of intervention

4.2.3 Analyze the feasibility of disseminating the intervention in the following strategies:

4.2.3.1 Poster / leaflet distribution

4.2.3.2 Social Media *Posts*

5. MATERIALS AND METHODS

This is an intervention study, a randomized controlled trial with PLWHA, divided into two groups. One of the groups will be presented with information about HPV and the HPV vaccine, prepared based on the Protection Motivation Theory (PMT). Another group will receive a clipping of information from the page maintained by the Ministry of Health dedicated to informing the population about HPV and the HPV vaccine . The materials for both groups will be delivered through two *websites* aimed at mobile devices (*mobile friendly*) with a common visual identity for the project. The "i-HPV" *website* , which will be displayed to the intervention group, and the "MS-HPV" *website , which will be displayed to the control group.*

5.1 SELECTION OF PARTICIPANTS

Recruitment will be carried out through two strategies: dissemination of printed invitations (posters and pamphlets) and *online invitations* (*posts* on social media).

5.1.1 Printed invitations: Made possible by making pamphlets and posters available at locations that assist the key study population in the city of Salvador, Bahia.

5.1.2 Online invitations: For the free publication of posts on social media by institutions and organizations with good visibility and reputation aimed at disseminating issues of interest to PLWHA.

5.2 INCLUSION CRITERIA

i. Living with HIV/ AIDS.
ii. Age between 18 and 45 years.
iii. Consent to participate in the study.

5.3 EXLUSION CRITERIA

i. Having taken any dose of the HPV vaccine.

Participants will be allocated in a 1:1 ratio using a computer algorithm. To prevent the allocation order from being discovered, the algorithm randomly allocates participants to one of the groups, but ensures that the difference in the number of participants in each group is no greater than five. Furthermore, given that the participant will likely access the system alone, we understand that this strategy will minimize the possibility of discovering the pattern used in the allocation and the chances of the participant circumventing the randomization.

5.4 THE INTERVENTION

Immediately after randomization, participants in the intervention group will be directed to the i-HPV *website* and will receive information about HPV and the HPV vaccine. This *website* will be developed in a *mobile version* , but it will also be accessible from *desktops* . The content will be produced based on the theoretical basis of the TMP and will later be revised, based on improvements identified after conducting a focus group with PLWHA and/or workers from institutions aimed at assisting this key population. For this group, the *website* will be developed in four sequential sections, with simple, accessible language and a design that follows the trend of intuitive scrolling on screens, typical of *smartphones* . The four sections are:

i. <u>Learn more about HPV</u> : This session included information on HPV prevalence, transmission, HPV-related diseases, and increased risk for PLWHA. **Perceived severity** and **perceived vulnerability were used as theoretical constructs in this section** .

ii. <u>Learn about the HPV vaccine</u> : This session provides information about the HPV vaccine, its effectiveness, recommendations for key populations, and testimonials focusing on the reasons why people living with HIV should get vaccinated. The theoretical construct used is the **effectiveness of the response** , in this case, of vaccination.

iii. <u>Frequently Asked Questions</u> : This is a question and answer session used to provide information related to barriers and concerns about HPV and the HPV vaccine obtained from previous research and the focus group conducted prior to the intervention. Here, the **response cost variable will be explored** .

iv. Get vaccinated : This session provides information on vaccination sites and the documents required to obtain the vaccine. At this stage, the construct of **self-efficacy** is explored, as the participant will be led to evaluate their intention to get vaccinated within a certain period of time .

5.5 THE CONTROL GROUP

Participants in the control group will be redirected to the MS-HPV *website immediately after randomization. This website will also present information about HPV and the HPV vaccine, but it will be obtained from the website* http://saude.gov.br/saude-de-az/hpv , which is dedicated to informing the general population about HPV and is maintained by the MS. To avoid major disparities in content between the groups, information from this website will be cut to a new page developed for the project. The content will be reproduced in an attempt to maintain correspondence with the topics covered on the intervention group page. Thus, the following sections will be reproduced: “What is HPV”, “Signs and Symptoms”, “Prevention” and “Questions and Answers”. This version will include the same content from the “Get Vaccinated” section, presented to the intervention group.

5.6 DATA COLLECTION

In both iHPV and MS-HPV, as soon as the participant starts browsing the “Get vaccinated” section, they will be asked about their intention to get vaccinated in the next three months. Their response will allow us to capture the primary outcome of the intervention. It also serves as a trigger for the start of the self-reported data collection phase.

It will be done in two ways: automatic collection via browsing data and questionnaire. In automatic collection, the origin and time of access to the pages will be recorded. Through questionnaires, sociodemographic data , intention to get vaccinated, evaluation of the intervention, and reasons for not getting vaccinated will be collected. All data will be collected electronically and saved in a database with restricted access to researchers.

6. ANALYSIS PROPOSAL

6.1 VARIABLES

The variables and the objectives they will respond to are described below.

6.1.1 Viability of invitations

It will be measured by the access rate in each dissemination strategy. The viability of physical invitations (VCF) will be measured by the ratio between the number of accesses to the P0 home page originated from posters and pamphlets (nP0PP) and the estimated number of services to PLWHA (nA) carried out at the service locations, during the recruitment period.

0

=

online invitations (VCO), the ideal situation would be to measure the number of accesses to P0 originated from *online posts* (nP0PO) in relation to the number of views of these same *posts* . This situation is not possible since the dissemination will be done via free advertisements and in accounts not managed by the project. Therefore, we will consider as the denominator the total number of followers, on the last day of recruitment, on the social media of the institutions listed in item 4.1.2 where the *posts* should be published (nS).

= 0

This way, it will be possible to compare strategies, evaluating their respective performance in recruiting participants.

6.1.2 Acceptability

It will be expressed by the percentage of satisfied participants in the three dimensions of analysis:

a) Information quality
b) Website quality
c) Usefulness of intervention

And by the indicators below:

d) Abandonment rate: Ratio between the number of participants who enter and leave without any interaction and the total number of accesses to the page.
e) Average dwell time, per page and per section

6.1.3 Effectiveness

The effectiveness of the intervention (E) will be obtained by the percentage of participants who declared their intention to get vaccinated. Measured by the ratio between the sum of the number of participants who declare that they will or intend to get vaccinated (nV) and the number of participants in the groups randomized to their respective *websites* (nP).

$$= \quad \frac{\quad}{\quad} \times 100$$

6.2 DATA ANALYSIS METHODOLOGY

A descriptive analysis of the frequency distribution of the main outcome variables and other variables will be performed. Continuous variables will be expressed as mean ± standard deviation with a 95% confidence interval (CI), and categorical variables will be expressed as frequencies and percentages. To assess the effects of the intervention on the intention to get vaccinated (effectiveness), the groups will initially be assessed for sample homogeneity in relation to demographic and health-related characteristics. Differences between the groups studied will be calculated using *Student 's t-test.* for quantitative variables and, for categorical variables, the comparison between groups will be performed using the Chi -square test. The logistic regression model will be used to compare the study groups in all outcomes, adjusting for potential confounding variables . Statistical analyses will be performed using the *software* Stata 12. P-values <0.05 will be considered statistically significant.

6.2.1 Primary outcome

Percentage of PLWHA intending to receive the HPV vaccine.

6.2.2 Secondary outcome

Vaccination initiation rate (at least one dose of vaccine) among PLWHA and complete vaccination rate (three doses of vaccine).

6.2.3 Sample size

The goal will be to enroll 836 to 650 participants (assuming a 30% to 10% dropout rate) or 586 evaluable participants. By obtaining a sample of 293 evaluable patients per group and adopting a type I error probability (alpha) of 5%, we will have 80% power to detect a difference of 10% (20% vs. 30%) or greater between the intervention group and the control group.

7. SCHEDULE

	DESCRIPTION
**2021**	Submission to CEP (February)
	Finalize prototype (April)
	Focus Group Discussion (May)
	Prototype review and adjustments after focus group discussion (May)
**2022**	Data collection (January to June)
	Data analysis (October to November)
	Preparation of the thesis and article (November to December)

8. BUDGET

The project will have a relatively low cost, therefore, it will be executed with the advisor's own resources and the CNPq bench fee. Equipment (computers, tablets, etc.) and LEMB software will be used to conduct data analysis.

**Item**	**Amount**	**Total**
Ream of paper	1	57.90
Web Designer Consulting	40hs	1,800.00
Social Communication Specialist Consulting	40hs	1,800.00
**TOTAL**		**R$ 3,657.90**

9. ETHICAL CONSIDERATIONS

All Guidelines and Regulatory Standards for Research Involving Human Beings - Resolution No. 466/2012 of the National Health Council will be followed. The principles of bioethics (autonomy, non-maleficence, beneficence and justice) will be respected. (CNS, 2012) . For the purposes of this study, research participants will be informed about the objectives and content of the research in accessible language on an *online form* . Therefore, it will not be possible to apply a signed version of the Free and Informed Consent Form (FICF). They will also be informed that participation in the study is completely voluntary and that consent to participate in the study may be withdrawn at any time. No participant may enter the study before consent has been obtained

The study protocol and other relevant documents will be submitted to the Research Ethics Committee for collegiate evaluation and approval. The database will be created without information about the identity of the study participants, therefore all analyses will be performed anonymously and with the privacy of the subjects included in the research guaranteed. The information collected will be used only for the purpose of the study and the results found will be published in scientific journals in the area.

9.1 ASSESSMENT OF RISKS AND BENEFITS:

9.1.1 Risks

Risk of embarrassment with some questions. However, this is mitigated by the fact that the participant will be able to do so in privacy since it is an *online questionnaire* .

9.1.2 Benefits

Benefit for the individual who, by participating in the study, may choose to receive the vaccine. Secondarily, the individual, being at the vaccination center, also tends to update the vaccination card, receiving any doses that may still be pending.

Identification of a more effective approach to promoting the HPV vaccine among PLWHA.

In addition, the study will generate a product that will be freely available for use and adaptation: the iHPV intervention . In addition, the work provides a guide for the production of relevant and efficient information since it will identify the TMP constructs and content related to HPV and the HPV vaccine that most captured the attention of the key population.

10. REFERENCES

ABRAMOWITZ, L. *et al.* Human papillomavirus genotype distribution in anal cancer in France: the EDiTH V study. **International Journal of Cancer** , vol. 129, no. 2, p. 433–439, 2011.

ABREU, M.N.S. *et al.* Knowledge and perception about HPV in the population over 18 years of age in the city of Ipatinga, MG, Brazil. **Ciências & Saúde Coletiva** , v. 23, n. 3, p. 849–860, 2018.

ANDRADE, J. *et al.* **SBIm/SBI Immunization Guide – HIV/AIDS 2016-2017** . São Paulo: [sn]. Available at: <https://sbim.org.br/publicacoes/guias/567-guia-de-imunizacao-sbim-sbi -hiv-aids-2016-2017>.

ARBYN, M. *et al.* EUROGIN 2011 roadmap on prevention and treatment of HPV-related diseases. **International Journal of Cancer** , vol. 131, no. 9, p. 1969–1982, 2012.

BEACHLER, DC *et al.* Incidence and risk factors of HPV-related and HPV-unrelated Head and Neck Squamous Cell Carcinoma in HIV-infected individuals. **Oral Oncology** , vol. 50, no. 12, p. 1169–1176, 2014.

BLOMBERG, M. *et al.* Genital warts and risk of cancer: A Danish study of nearly 50,000 patients with genital warts. **Journal of Infectious Diseases** , vol. 205, n. 10, p. 1544–1553, 2012.

BOSCH, FX *et al.* The causal relationship between human papillomavirus and cervical cancer. **Journal of Clinical Pathology** , vol. 55, n. 4, p. 244–265, 2002.

BRAZIL. Technical report on the expansion of the supply of human papillomavirus 6, 11, 16 and 18 (recombinant) vaccines – quadrivalent HPV and meningococcal C (conjugate) vaccine. **Ministry of Health. Health Surveillance Secretariat. Department of Communicable Disease Surveillance. General Coordination of the National Immunization Program.** , v. 18, p. 1–39, 2018.

___. Technical report on the supply of human papillomavirus 6, 11, 16 and 18 vaccine (recombinant) – quadrivalent HPV vaccine. **Ministry of Health. Health Surveillance Secretariat. Department of Surveillance of Communicable Diseases. General Coordination of the National Immunization Program.** , p. 27, 2019.

BRUNI, L. *et al.* **Human Papillomavirus and Related Diseases ReportICO/IARC Information Center on HPV and Cancer (HPV Information Center)** . [sl: sn]. Available at: <https://www.hpvcentre.net/statistics/reports/XWX.pdf>.

CAMERINI, AL *et al.* Using protection motivation theory to predict intention to adhere to official MMR vaccination recommendations in Switzerland. **SSM - Population Health** , v. 7, no. November 2018, p. 100321, 2019.

CDC. **HPV and Cancer** . Available at: <https://www.cdc.gov/cancer/hpv/statistics/index.htm>. Accessed on: March 13, 2019.

CETIC.BR. **ICT households. Research on the Use of TechnologiesResearch on the use of information and communication technologies in Brazilian households: ICT households 2017** . São Paulo: [sn].

CHATURVEDI, AK *et al.* Risk of human papillomavirus-associated cancers among persons with AIDS. **Journal of the National Cancer Institute** , vol. 101, no. 16, p. 1120–1130, 2009.

COELHO, P.L.S. *et al.* Safety of human papillomavirus 6, 11, 16 and 18 (recombinant) vaccine: systematic review and meta-analysis. **Revista Paulista de Pediatria** , v. 33, n. 4, p. 474–482, 2015.

DENNY, L. *et al.* Safety and immunogenicity of the HPV-16/18 AS04-adjuvanted vaccine in HIV-positive women in South Africa: A partially-blind randomized placebo-controlled study. **Vaccine** , vol. 31, no. 48, p. 5745–5753, 2013.

DENNY, LA *et al.* Human papillomavirus, human immunodeficiency virus and immunosuppression. **Vaccine** , vol. 30, no. SUPPL.5, p. F168–F174, 2012.

DREYER, G. Clinical implications of the interaction between HPV and HIV infections. **Best Practice and Research: Clinical Obstetrics and Gynaecology** , vol. 47, p. 95– 106, 2018.

FONTENOT, HB *et al.* Increasing HPV vaccination and eliminating barriers: Recommendations from young men who have sex with men. **Vaccine** , vol. 34, no. 50, p. 6209–6216, 2016.

FRISCH, M. Human Papillomavirus-Associated Cancers in Patients With Human Immunodeficiency Virus Infection and Acquired Immunodeficiency Syndrome. **Journal of the National Cancer Institute** , vol. 92, no. 18, p. 1500–1510, 2000.

GAINFORTH, H.L.; CAO, W.; LATIMER-CHEUNG, AE Determinants of human papillomavirus (HPV) vaccination intent among three Canadian target groups. **Journal of Cancer Education** , vol. 27, no. 4, p. 717–724, 2012.

GAINFORTH, H.L.; LATIMER, AE Risky business: Risk information and the moderating effect of message frame and past behavior on women's perceptions of the Human Papillomavirus vaccine. **Journal of Health Psychology** , vol. 17, no. 6, p. 896–906, 2012.

GIACOMET, V. *et al.* Safety and immunogenicity of a quadrivalent human papillomavirus vaccine in HIV-infected and HIV-negative adolescents and young adults. **Vaccine** , vol. 32, no. 43, p. 5657–5661, 2014.

HESSOL, NA *et al.* Anal intraepithelial neoplasia in a multisite study of HIV-infected and high-risk HIV-uninfected women. **AIDS** , vol. 23, no. 1, p. 59–70, 2009.

HOULIHAN, CF *et al.* HPV infection and increased risk of HIV acquisition. A systematic review and meta-analysis. **Europe PMC Funders Manuscripts** , vol. 26, no. 17, p. 1–18, 2013.

IARC. **World Cancer Day 2019 IARC: “HPV vaccination is safe, effective, and critical for eliminating cervical cancer"** . Available at: <https://www.iarc.fr/wp-content/uploads/2019/02/pr264_E.pdf> . Accessed on: 19 Feb 2018.

INCA. **2018 Estimate: Cancer Incidence in Brazil** . Rio de Janeiro: [sn]. Available at: <https://www.inca.gov.br/sites/ufu.sti.inca.local/files//media/ document//estimativa-incidencia-de-cancer-no-brasil-2018.pdf>.

KAHN, JA *et al.* Immunogenicity and safety of the human papillomavirus 6, 11, 16, 18 vaccine in HIV-infected young women. **Clinical Infectious Diseases** , vol. 57, no. 5, p. 735–744, 2013.

KELLER, MJ *et al.* Risk of cervical precancer and cancer among HIV-infected women with normal cervical cytology and no evidence of oncogenic HPV infection. **JAMA - Journal of the American Medical Association** , v. 308, no. 4, p. 362–369, 2012.

KOJIC, EM *et al.* Immunogenicity and safety of the quadrivalent human papillomavirus vaccine in HIV-1-infected women. **Clinical Infectious Diseases** , vol. 59, no. 1, p. 127–135, 2014.

LEONCINI, E. *et al.* Adult height and head and neck cancer: A pooled analysis within the INHANCE Consortium. **Head and Neck** , vol. 36, no. 10, p. 1391, 2014.

LEVIN, MJ *et al.* Safety and immunogenicity of a quadrivalent human papillomavirus (types 6, 11, 16, and 18) vaccine in HIV-infected children 7 to 12 years old. **Journal of Acquired Immune Deficiency Syndromes** , vol. 55, n. 2, p. 197–204, 2010.

LEWIS, RM *et al.* Prevalence of Genital Human Papillomavirus among Sexually Experienced Males and Females Aged 14-59 Years, United States, 2013-2014. **Journal of Infectious Diseases** , vol. 217, no. 6, p. 869–877, 2018.

LIU, R. *et al.* Analysis of hepatitis B vaccination behavior and vaccination willingness among migrant workers from rural China based on protection motivation theory. **Human Vaccines and Immunotherapeutics** , vol. 12, no. 5, p. 1155–1163, 2016.

LOOKER, KJ *et al.* Evidence of synergistic relationships between HIV and Human Papillomavirus (HPV): systematic reviews and meta-analyses of longitudinal studies of HPV acquisition and clearance by HIV status, and of HIV acquisition by HPV status. **Journal of the International AIDS Society** , vol. 21, no. 6, 2018.

MACHALEK, DA *et al.* Anal human papillomavirus infection and associated neoplastic lesions in men who have sex with men: A systematic review and meta-analysis. **The Lancet Oncology** , vol. 13, no. 5, p. 487–500, 2012.

MACHALEK, DA *et al.* Very Low Prevalence of Vaccine Human Papillomavirus Types Among 18- to 35-Year Old Australian Women 9 Years Following

Implementation of Vaccination. **The Journal of Infectious diseases** , vol. 217, p. 1590–1600, 2018.

MADDUX, J.E.; ROGERS, RW Protection motivation and self-efficacy: A revised theory of fear appeals and attitude change. **Journal of Experimental Social Psychology** , vol. 19, no. 5, p. 469–479, 1983.

MARTEL, C. DE *et al.* Worldwide burden of cancer attributable to HPV by site, country and HPV type. **International Journal of Cancer** , vol. 141, no. 4, p. 664–670, 2017.

MCREE, AL *et al.* Outsmart HPV: Acceptability and short-term effects of a web-based HPV vaccination intervention for young adult gay and bisexual men. **Vaccine** , vol. 36, no. 52, p. 8158–8164, 2018.

MENDES LOBÃO, W. *et al.* Low coverage of HPV vaccination in the national immunization program in Brazil: Parental vaccine refusal or barriers in health-service based vaccine delivery? **PloS one** , v. 13, no. 11, p. e0206726, 2018.

MOREIRA, ED *et al.* Knowledge and attitudes about human papillomavirus, Pap smears, and cervical cancer among young women in Brazil: Implications for health education and prevention. **International Journal of Gynecological Cancer** , vol. 16, no. 2, p. 599–603, 2006.

MOSCICKI, AB *et al.* Human papillomavirus infection and abnormal cytology of the anus in HIV-infected and uninfected adolescents. **AIDS** , vol. 17, no. 3, p. 311–320, 2003.

NOWAK, RG *et al.* Increases in human papillomavirus detection during early HIV infection among women in Zimbabwe. **Journal of Infectious Diseases** , vol. 203, no. 8, p. 1182–1191, 2011.

NYGÅRD, M. *et al.* Evaluation of the long-term anti-human papillomavirus 6 (HPV6), 11, 16, and 18 immune responses generated by the quadrivalent HPV vaccine. **Clinical and Vaccine Immunology** , vol. 22, no. 8, p. 943–948, 2015.

NYITRAY, AG The epidemiology of anal human papillomavirus infection among women and men having sex with women. **Sexual Health** , vol. 9, no. 6, p. 538–546, 2012.

PEREIRA, RGV *et al.* The influence of knowledge on attitudes towards the Human Papillomavirus vaccine: a randomized clinical trial. **ABCS Health Sciences** , v. 41, n. 2, p. 78–83, 2016.

PERNOT, S. *et al.* Immunity and squamous cell carcinoma of the anus: Epidemiological, clinical and therapeutic aspects. **Clinics and Research in Hepatology and Gastroenterology** , vol. 38, no. 1, p. 18–23, 2014.

POLLOCK, KGJ *et al.* Reduction of low- and high-grade cervical abnormalities associated with high uptake of the HPV bivalent vaccine in Scotland. **British Journal**

of Cancer , vol. 111, no. 9, p. 1824–1830, 2014.

POP-BRASIL. **Epidemiological study on the national prevalence of HPV infection** . [sl: sn].

REITER, PL *et al.* Increasing human papillomavirus vaccination among young gay and bisexual men: A randomized pilot trial of the outsmart HPV intervention. **LGBT Health** , vol. 5, no. 5, p. 325–329, 2018.

ROGERS, RW A Protection Motivation Theory of Fear Appeals and Attitude Change1. **The Journal of Psychology** , vol. 91, no. 1, p. 93–114, 1975.

RUBIN, MA *et al.* Detection and typing of human papillomavirus DNA in penile carcinoma: Evidence for multiple independent pathways of penile carcinogenesis. **American Journal of Pathology** , vol. 159, no. 4, p. 1211–1218, 2001.

SCHILLER, JT; CASTELLSAGUÉ, X.; GARLAND, SM A review of clinical trials of human papillomavirus prophylactic vaccines. **Vaccine** , vol. 30, no. SUPPL.5, p. F123–F138, 2012.

SCHIM VAN DER LOEFF, MF *et al.* HPV and anal cancer in HIV-infected individuals: a review. **Current HIV/AIDS reports** , vol. 11, no. 3, p. 250–262, 2014.

SCHWARZ, TF *et al.* A ten-year study of immunogenicity and safety of the AS04-HPV-16/18 vaccine in adolescent girls aged 10-14 years. **Human Vaccines and Immunotherapeutics** , vol. 15, no. 7–8, p. 1970–1979, 2019.

SHVETSOV, YB *et al.* Duration and Clearance of Anal Human Papillomavirus (HPV) Infection among Women: The Hawaii HPV Cohort Study. **Clinical Infectious Diseases** , vol. 48, no. 5, p. 536–546, 2009.

SILVA, LEL DA; OLIVEIRA, MLC DE; GALATO, D. Receptivity to the human papillomavirus vaccine: a systematic review. **Rev Panam Salud Publica** , v. 43, p. 1–9, 2019.

SMITH, MA *et al.* Fall in Genital Warts Diagnoses in the General and Indigenous Australian Population Following Implementation of a National Human Papillomavirus Vaccination Program: Analysis of Routinely Collected National Hospital Data. **The Journal of Infectious diseases** , vol. 211, p. 91–99, 2015.

TOZZI, AE *et al.* Comparison of Quality of Internet Pages on Human Papillomavirus Immunization in Italian and in English. **Journal of Adolescent Health** , vol. 46, no. 1, p. 83–89, 2010.

VELDHUIJZEN, NJ *et al.* The epidemiology of human papillomavirus infection in HIV-positive and HIV-negative high-risk women in Kigali, Rwanda. **BMC Infectious Diseases** , v. 11, no. 1, p. 333, 2011.

WHO. **Guide to Introducing HPV Vaccine Into National Immunization Programmes** . Available at: <www.who.int/immunization/documents>. Accessed on:

March 14, 2019.

___. **Weekly epidemiological record** . Available at: <https://www.who.int/immunization/policy/position_papers/hpv/en/>. Accessed on: March 13, 2019.

___. **Human papillomavirus (HPV) and cervical cancer** . Available at: <https://www.who.int/en/news-room/fact-sheets/detail/human-papillomavirus-(hpv)-and-cervical-cancer#>. Accessed on: March 13, 2019.

WILKIN, T. *et al.* Safety and Immunogenicity of the Quadrivalent Human Papillomavirus Vaccine in HIV-1–Infected Men. **The Journal of Infectious Diseases** , vol. 202, no. 8, p. 1246–1253, 2010.

WILLIAMSON, A.-L. The Interaction between Human Immunodeficiency Virus and Human Papillomaviruses in Heterosexuals in Africa. **Journal of Clinical Medicine** , vol. 4, no. 4, p. 579–592, 2015.
